# Supplementary material for: Engineering and elucidation of the lipoinitiation process in nonribosomal peptide biosynthesis
Source: Nat Commun. 2021 Jan 12;12:296. doi: 10.1038/s41467-020-20548-8 (PMC7804268; doi:10.1038/s41467-020-20548-8)
Supplement: Supplementary file 1 — Supplementary Information [file 41467_2020_20548_MOESM1_ESM.pdf]

Supplementary information

Engineering and Elucidation of the Lipoinitiation Process in Nonribosomal Peptide Biosynthesis

Lin Zhong[1#], Xiaotong Diao[1#], Na Zhang[1#], Fengwei Li[1], Haibo Zhou[1], Hanna Chen[1], Xianping Bai[1], Xintong Ren[1], Youming Zhang[1]*, Dalei Wu[1,2]* & Xiaoying Bian[1]*

[1]Helmholtz International Lab for Anti-Infectives, Shandong University-Helmholtz Institute of Biotechnology, State Key Laboratory of Microbial Technology, Shandong University, Qingdao, Shandong 266237, China.

[2]Suzhou Research Institute, Shandong University, Suzhou, Jiangsu 215123, China.

[#]These authors contributed equally.

***Corresponding authors**

Youming Zhang (zhangyouming@sdu.edu.cn)
Dalei Wu (dlwu@sdu.edu.cn)
Xiaoying Bian (bianxiaoying@sdu.edu.cn)

Supplementary Tables

Supplementary Table 1. The ^{1}H-NMR (600 MHz) Data of **1d, 1e, 1f, 1g, 1h** in DMSO-d_6
Supplementary Table 2. The ^{13}C-NMR (150 MHz) Data of **1d, 1e, 1f, 1g, 1h** in DMSO-d_6
Supplementary Table 3. The ^{1}H (600 MHz) and ^{13}C NMR (150 MHz) Data of **2a** in DMSO-d_6
Supplementary Table 4. The ^{1}H (500 MHz) and ^{13}C NMR (125 MHz) Data of **7b**,**7c** in DMSO-d_6
Supplementary Table 5. Data collection and refinement statistics

Supplementary Figures

Supplementary Fig. 1. The *in vitro* biochemical experiments of dissected RzmA-Cs and HolA-Cs domains
Supplementary Fig. 2. Structural determination of rhizomide derivatives **1b-1h**
Supplementary Fig. 3. Structural determination of holrhizin derivatives
Supplementary Fig. 4. Comparison between RzmA and HolA Cs domains
Supplementary Fig. 5. Substrate selection of the RzmA-Cs mutants
Supplementary Fig. 6. Crystal structures of RzmA-Cs mutants in complex with the CoA substrate
Supplementary Fig. 7. Crystal structure of RzmA-Cs mutant H140V/R148A in complex with two substrates
Supplementary Fig. 8. A picture showing two types of RzmA-Cs H140V/R148A crystals growing in the same drop
Supplementary Fig. 9. Structure comparison of RzmA-Cs mutant H140V/R148A in different forms
Supplementary Fig. 10. The *in vitro* biochemical experiments of dissected RzmA-Cs to simulate *in vivo* conditions
Supplementary Fig. 11. ^{1}H NMR spectrum of **2a** in DMSO-d_6
Supplementary Fig. 12. ^{13}C NMR spectrum of **2a** in DMSO-d_6
Supplementary Fig. 13. ^{1}H-^{1}H COSY spectrum of **2a** in DMSO-d_6
Supplementary Fig. 14. HMBC spectrum of **2a** in DMSO-d_6
Supplementary Fig. 15. HSQC spectrum of **2a** in DMSO-d_6
Supplementary Fig. 16. ^{1}H NMR spectrum of **1d** in DMSO-d_6
Supplementary Fig. 17. ^{13}C NMR spectrum of **1d** in DMSO-d_6
Supplementary Fig. 18. ^{1}H-^{1}H COSY spectrum of **1d** in DMSO-d_6
Supplementary Fig. 19. HMBC spectrum of **1d** in DMSO-d_6
Supplementary Fig. 20. HSQC spectrum of **1d** in DMSO-d_6
Supplementary Fig. 21. ^{1}H NMR spectrum of **1e** in DMSO-d_6
Supplementary Fig. 22. ^{13}C NMR spectrum of **1e** in DMSO-d_6
Supplementary Fig. 23. ^{1}H NMR spectrum of **1f** in DMSO-d_6
Supplementary Fig. 24. ^{13}C NMR spectrum of **1f** in DMSO-d_6
Supplementary Fig. 25. ^{1}H NMR spectrum of **1g** in DMSO-d_6
Supplementary Fig. 26. ^{13}C NMR spectrum of **1g** in DMSO-d_6
Supplementary Fig. 27. ^{1}H NMR spectrum of **1h** in DMSO-d_6
Supplementary Fig. 28. ^{13}C NMR spectrum of **1h** in DMSO-d_6
Supplementary Fig. 29. ^{1}H NMR spectrum of **7b** in DMSO-d_6
Supplementary Fig. 30. ^{13}C NMR spectrum of **7b** in DMSO-d_6

Supplementary Fig. 31. ^{1}H NMR spectrum of **7c** in DMSO-d_6
Supplementary Fig. 32. ^{13}C NMR spectrum of **7c** in DMSO-d_6

Supplementary Tables

Supplementary Table 1. The ^{1}H (600 MHz) Data of **1d**, **1e**, **1f**, **1g** and **1h** in DMSO-d_6

	No.	**1d** (C8-RzmA)	**1e** (C10-RzmA)	**1f** (C12-RzmA)	**1g** (C14-RzmA)	**1h** (C16-RzmA)
FA	2a	2.14, m	2.12, m	2.12, m	2.12, m	2.12, m
	2b	2.11, m				
	3	1.48, m	1.48, m	1.48, m	1.48, m	1.48, m
	4	1.21, m	1.21, m[a]	1.21, m[a]	1.21, m[a]	1.21, m[a]
	5	1.21, m	1.21, m[a]	1.21, m[a]	1.21, m[a]	1.21, m[a]
	6	1.21, m	1.21, m[a]	1.21, m[a]	1.21, m[a]	1.21, m[a]
	7	1.21, m	1.21, m[a]	1.21, m[a]	1.21, m[a]	1.21, m[a]
	8	0.84, t (7.0)	1.21, m[a]	1.21, m[a]	1.21, m[a]	1.21, m[a]
	9		1.21, m[a]	1.21, m[a]	1.21, m[a]	1.21, m[a]
	10		0.84, t (7.1)	1.21, m[a]	1.21, m[a]	1.21, m[a]
	11			1.21, m[a]	1.21, m[a]	1.21, m[a]
	12			0.85, t (6.9)	1.21, m[a]	1.21, m[a]
	13				1.21, m[a]	1.21, m[a]
	14				0.85, t (6.9)	1.21, m[a]
	15					1.21, m[a]
	16					0.85, t (6.9)
Leu	2	4.50, m	4.49, m	4.50, m	4.50, m	4.50, m
	3a	1.58, m[a]	1.58, m[a]	1.59, m[a]	1.59, m[a]	1.59, m[a]
	3b	1.42, m	1.42, m	1.42, m	1.43, m	1.42, m
	4	1.58, m[a]	1.58, m[a]	1.59, m[a]	1.59, m[a]	1.59, m[a]
	5	0.90, d (6.5)	0.90, d (6.4)	0.90, d (6.4)	0.90, d (6.4)	0.90, d (6.4)
	6	0.81, d (6.5)	0.81, d (6.4)	0.81, d (6.4)	0.81, d (6.5)	0.81, d (6.5)
	NH	8.33, d (8.0)	8.29, d (8.2)	8.30, d (8.0)	8.30, d (8.2)	8.30, d (8.2)
Thr	2	4.46, dd (2.3, 9.1)	4.46, dd (2.2, 9.0)	4.46, dd (1.8, 9.0)	4.46, dd (2.2, 9.0)	4.46, dd (2.1, 9.0)
	3	5.20, m	5.21, dq (2.2, 6.4)	5.22, dq (1.8, 6.5)	5.22, dq (2.2, 6.5)	5.22, dq (2.2, 6.5)
	4	1.00, d (6.4)	1.00, d (6.5)	1.00, d (6.5)	1.00, d (6.5)	1.00, d (6.5)
	NH	7.86, d (8.8)	7.77, d (9.0)	7.77, d (9.0)	7.77, d (9.0)	7.77, d (9.0)
Tyr	2	4.37, m	4.38, ddd (5.1, 8.9,13.3)	4.38, ddd (4.9, 8.9,13.4)	4.38, ddd (5.0, 8.8,13.4)	4.38, ddd (5.0, 8.9,13.4)
	3a	2.98, dd (4.9, 14.4)	2.98, dd (5.1, 14.4)	2.98, dd (4.9, 14.4)	2.98, dd (4.9, 14.4)	2.98, dd (4.9, 14.3)
	3b	2.83, dd (9.0, 14.4)	2.83, dd (8.9, 14.4)	2.83, dd (8.9, 14.4)	2.83, dd (8.8, 14.4)	2.83, dd (8.9, 14.3)
	5/9	7.02, d (8.4)	7.02, d (8.5)	7.02, d (8.4)	7.02, d (8.5)	7.02, d (8.5)
	6/8	6.63, d (8.4)	6.63, d (8.5)	6.63, d (8.5)	6.63, d (8.5)	6.63, d (8.5)
	NH	7.46, d (7.2)	7.35, d (7.4)	7.36, d (6.9)	7.36, d (7.7)	7.36, d (7.8)
	OH	9.27, s	9.20, brs	9.19, brs	9.19, brs	9.19, s
Ala1	2	4.04, m	4.04, dq (5.0, 7.0)	4.04, dq (4.3, 7.0)	4.04, dq (5.8, 7.0)	4.04, dq (4.2, 7.0)
	3	1.29, d (6.9)	1.29, d (7.0)	1.30, d (7.0)	1.30, d (7.0)	1.30, d (7.0)
	NH	8.15, d (4.7)	8.11, d[a] (5.0)	8.10, d[a] (4.3)	8.10, d[a] (5.8)	8.10, d[a] (4.2)
Ala2	2	4.28, m	4.28, dq (7.0, 7.1)	4.29, dq (7.0, 7.1)	4.29, dq (7.0, 7.1)	4.29, dq (7.0, 7.1)
	3	1.22, d (7.3)	1.22, d (7.0)	1.22, d (7.0)	1.22, d (7.0)	1.22, d (7.0)
	NH	8.64, d (7.0)	8.63, d (7.1)	8.64, d (7.1)	8.64, d (7.1)	8.64, d (7.1)
Ala3	2	4.20, m	4.19, dq (6.2, 8.2)	4.20, dq (4.3, 7.4)	4.20, dq (5.8, 7.4)	4.20, dq (4.2, 7.4)
	3	1.23, d (7.1)	1.21, d (6.2)	1.21, d (7.4)	1.21, d (7.4)	1.21, d (7.4)
	NH	8.13, d (7.7)	8.10, d (8.2)	8.10, d[a] (4.3)	8.10, d[a] (5.8)	8.10, d[a] (4.2)
Val	2	4.13, dd (7.6, 8.8)	4.14, dd (7.6, 8.9)	4.14, dd (7.2, 8.8)	4.14, dd (7.6, 8.9)	4.14, dd (7.7, 8.9)
	3	1.83, m	1.82, m	1.82, m	1.82, m	1.82, m
	4	0.78, d (6.7)	0.78, d (6.8)	0.78, d (6.7)	0.78, d (6.7)	0.78, d (6.7)
	5	0.72, d (6.7)	0.71, d (6.7)	0.71, d (6.8)	0.71, d (6.7)	0.71, d (6.7)
	NH	7.34, d (8.9)	7.32, d (8.9)	7.31, d (8.8)	7.31, d (9.0)	7.31, d (9.0)

Supplementary Table 2. The ^{13}C NMR (150 MHz) Data of **1d**, **1e**, **1f**, **1g** and **1h** in DMSO-d_6

	No.	**1d**	**1e**	**1f**	**1g**	**1h**
FA	1	172.9, C	173.0, C	173.0, C	173.0, C	173.0, C
	2	35.1, CH_2	35.1, CH_2	35.1, CH_2	35.1, CH_2	35.1, CH_2
	3	25.4, CH_2	25.4, CH_2	25.4, CH_2	25.4, CH_2	25.3, CH_2
	4	28.4, CH_2	28.5, CH_2	28.5, CH_2	28.5, CH_2	28.5, CH_2
	5	28.4, CH_2	28.6, CH_2	28.7, CH_2	28.7, CH_2	28.7, CH_2
	6	31.2, CH_2	28.9, CH_2	29.0, CH_2	29.0, CH_2	28.9, CH_2
	7	22.0, CH_2	28.7, CH_2	29.0, CH_2	29.0, CH_2	29.0, CH_2
	8	14.0, CH_3	31.3, CH_2	29.0, CH_2	29.0, CH_2	29.0, CH_2
	9		22.1, CH_2	28.7, CH_2	29.0, CH_2	29.0, CH_2
	10		14.0, CH_3	31.3, CH_2	29.0, CH_2	29.0, CH_2
	11			22.1, CH_2	28.7, CH_2	29.0, CH_2
	12			14.0, CH_3	31.3, CH_2	29.0, CH_2
	13				22.1, CH_2	28.7, CH_2
	14				14.0, CH_3	31.3, CH_2
	15					22.1, CH_2
	16					14.0, CH_3
Leu	1	172.7, C	172.7, C	172.7, C	172.7, C	172.7, C
	2	50.7, CH	50.7, CH	50.7, CH	50.7, CH	50.7, CH
	3	39.8, CH_2	39.2, CH_2	39.2, CH_2	39.2, CH_2	39.2, CH_2
	4	24.0, CH	24.0, CH	24.0, CH	24.0, CH	24.0, CH
	5	23.2, CH_3	23.2, CH_3	23.2, CH_3	23.2, CH_3	23.2, CH_3
	6	21.0, CH_3	21.0, CH_3	21.0, CH_3	21.0, CH_3	21.0, CH_3
Thr	1	168.2, C	168.2, C	168.2, C	168.2, C	168.2, C
	2	55.2, CH	55.1, CH	55.2, CH	55.2, CH	55.2, CH
	3	70.0, CH	70.1, CH	70.1, CH	70.1, CH	70.1, CH
	4	15.5, CH_3	15.5, CH_3	15.5, CH_3	15.5, CH_3	15.5, CH_3
Tyr	1	170.9, C	170.9, C	170.9, C	170.9, C	170.9, C
	2	54.8, CH	54.7, CH	54.8, CH	54.8, CH	54.8, CH
	3	36.0, CH_2	36.0, CH_2	36.0, CH_2	36.0, CH_2	36.0, CH_2
	4	127.1, C	127.1, C	127.1, C	127.1, C	127.1, C
	5/9	130.0, CH	130.0, CH	130.0, CH	130.0, CH	130.0, CH
	6/8	115.0, CH	115.0, CH	115.0, CH	115.0, CH	115.0, CH
	7	160.0, C	155.9, C	155.9, C	155.9, C	155.9, C
Ala1	1	172.7, C	172.7, C	172.7, C	172.7, C	172.7, C
	2	48.9, CH	48.9, CH	48.9, CH	48.9, CH	48.9, CH
	3	17.2, CH_3	17.2, CH_3	17.2, CH_3	17.2, CH_3	17.2, CH_3
Ala2	1	171.8, C	171.8, C	171.8, C	171.8, C	171.8, C
	2	48.8, CH	48.8, CH	48.8, CH	48.8, CH	48.8, CH
	3	16.3, CH_3	16.3, CH_3	16.3, CH_3	16.3, CH_3	16.3, CH_3
Ala3	1	171.7, C	171.7, C	171.7, C	171.7, C	171.7, C
	2	49.4, CH	49.4, CH	49.4, CH	49.4, CH	49.4, CH
	3	17.5, CH_3	17.5, CH_3	17.5, CH_3	17.5, CH_3	17.5, CH_3
Val	1	169.4, C	169.3, C	169.3, C	169.3, C	169.3, C
	2	57.7, CH	57.6, CH	57.6, CH	57.6, CH	57.6, CH
	3	30.3, CH	30.3, CH	30.3, CH	30.3, CH	30.3, CH
	4	18.9, CH_3	18.8, CH_3	18.8, CH_3	18.8, CH_3	18.8, CH_3
	5	18.0, CH_3	18.0, CH_3	18.0, CH_3	18.0, CH_3	18.0, CH_3

Supplementary Table 3. The ^{1}H (600 MHz) and ^{13}C NMR (150 MHz) Data of **2a** in DMSO-d_6

	No.	δ_C	δ_H	Mult. (J in Hz)
HAc	1	169.3, C		
	2	22.4, CH_3	1.84	s
Val	1	170.8, C		
	2	57.9, CH	4.06	dd (7.3, 8.3)
	3	30.0 CH	1.86	m
	4	19.1, CH_3	0.73	d (7.3)
	5	18.0, CH_3	0.74	d (7.3)
	2-NH		7.79	d (8.3)
Phe	1	170.9, C		
	2	53.5, CH	4.55	m
	3a	37.2, CH_2	3.05	dd (4.2, 14.2)
	3b		2.79	dd (10.2, 14.2)
	4	137.7, C		
	5/9	129.1, CH	7.23	m[a]
	6/8	127.9, CH	7.23	m[a]
	7	126.1, CH	7.17	t (6.7)
	2-NH		7.95	d (8.0)
Glu	1	171.2, C		
	2	51.5, CH	4.17	m
	3a	27.3, CH_2	1.95	m
	3b		1.78	m[a]
	4a	31.5, CH_2	2.25	m
	4b		2.18	m
	5	172.9, C		
	2-NH		8.10	d (7.8)
	5-COOH		12.60	brs
Ile1	1	170.7, C		
	2	56.6, CH	4.20	m
	3	36.6, CH	1.68	m
	4a	24.2, CH_2	1.39	m[a]
	4b		1.07	m
	5	10.9, CH_3	0.77	t (7.3)
	6	15.2, CH_3	0.84	d (7.1)
	2-NH		7.83	d (9.1)
Ala	1	171.9, C		
	2	47.9, CH	4.36	m
	3	17.9, CH_3	1.19	d (7.0)
	2-NH		8.08	d (7.3)
Ile2	1	172.6, C		
	2	56.1, CH	4.16	m
	3	36.4, CH	1.78	m[a]
	4	24.5, CH_2	1.39	m[a]
	4b		1.18	m[a]
	5	11.1, CH_3	0.82	t (6.6)
	6	15.3, CH_3	0.84	d (7.1)
	2-NH		7.81	d (8.3)
	1-COOH		12.60	brs

[a] overlapped

Supplementary Table 4. The ^{1}H (500 MHz) and ^{13}C NMR (125 MHz) Data of **7b**,**7c** in DMSO-d_6

Position	**7b**		**7c**	
	δ_C	δ_H (J in Hz)	δ_C	δ_H (J in Hz)
1	167.7, C		167.6, C	
2	123.3, CH	6.19, d (15.8)	123.3, CH	6.18, d (15.9)
3	143.1, CH	6.40, dd (4.3, 15.8)	143.0, CH	6.40, dd (4.3, 15.9)
4	44.8, CH	4.38, m	44.7, CH	4.38, m
5	18.5, CH_3	1.24, d[a]	18.5, CH_3	1.24, d[a]
6	171.1, C		171.1, C	
7	51.2, CH	4.34, m	51.2, CH	4.33, m
8a	42.4, CH_2	1.83, m	42.4, CH_2	1.83, m
8b		1.58, m		1.58, m
9	66.9, CH	3.57, m	67.0, CH	3.57, m
10a	39.2, CH_2	1.48, m[a]	39.2, CH_2	1.47, m[a]
10b		1.37, m		1.37, m
11a	39.9, CH_2	3.03, m	39.9, CH_2	3.02, m
11b		2.98, m		2.98, m
12	169.5, C		169.5, C	
13	57.9, CH	4.18, dd (3.7, 8.6)	57.9, CH	4.18, dd (3.7, 8.6)
14	66.6, CH	3.94, m	66.6, CH	3.93, m
15	19.9, CH_3	0.99, d (6.2)	19.9, CH_3	0.99, d (6.2)
16	172.6, C		172.5, C	
17	35.3, CH_2	2.16, m	35.3, CH_2	2.16, m
18	25.4, CH_2	1.48, m[a]	25.4, CH_2	1.47, m[a]
19	28.5, CH_2	1.24, m[a]	28.6, CH_2	1.24, m[a]
20	28.6, CH_2	1.24, m[a]	28.7, CH_2	1.24, m[a]
21	31.2, CH_2	1.24, m[a]	28.9, CH_2	1.24, m[a]
22	22.1, CH_2	1.24, m[a]	28.8, CH_2	1.24, m[a]
23	14.0, CH_3	0.86, t (6.7)	31.3, CH_2	1.24, m[a]
24			22.1, CH_2	1.24, m[a]
25			14.0, CH_3	0.85, t (6.5)
4-NH		8.64, brs		8.67, brs
7-NH		7.64, brs		7.65, d (7.6)
9-OH		4.67, brs		4.74, brs
11-NH		7.40, t (5.8)		7.42, t (6.1)
13-NH		7.69, d (8.5)		7.69, d (8.8)
14-OH		4.81, brs		4.74, brs

[a] Overlapped

Supplementary Table 5. Data collection and refinement statistics

	RzmA-Cs WT	R148A	R148A+C8-CoA	H140V/R148A	H140V/R148A +C8-CoA	H140V/R148A+C8-CoA+Leu-SNAC	H140V/R148A product-released
**PDB code**	7C1H	7C1K	7C1L	7C1P	7C1R	7C1S	7C1U
**Data collection**							
Space group	P1	P1	P212121	P1	P212121	P212121	P212121
Cell dimensions							
a, *b*, *c* (Å)	60.05,88.76,97.88	59.97,89.70, 98.35	52.91,72.33,108.54	60.64,89.65,98.07	52.77,72.09,108.28	52.83,72.39,108.58	52.56,58.78,134.06
α, β, γ (°)	67.30,89.90,89.92	113.22,90.09,89.92	90,90,90	66.89,90.02.90.25	90,90,90	90,90,90	90 90 90
Resolution (Å)	50-2.30 (2.34-2.3)*	50-2.80(2.85-2.80)	50-1.85(1.95-1.85)	50-2.60(2.64-2.60)	50-1.7(1.73-1.70)	50-2.6 (2.62-2.58)	50-1.40(1.42-1.40)
R_{sym} or R_{merge}	3.9 (52.6)	6.7 (36.6)	6.8 (53.2)	4.4 (36.3)	5.6 (86.6)	15.2 (76.2)	5.6 (89.7)
I / δI	34.9 (2.4)	16.8 (2.7)	17.8 (2.8)	28.7 (3.2)	46.4 (2.7)	16.4 (2.8)	45.1 (2.3)
Completeness (%)	94.7 (96.5)	94.8(88.7)	99.8 (98.7)	94.8 (95.5)	99.8 (99.0)	99.9 (100.0)	97.9(92.9)
Redundancy	3.6 (3.6)	3.6(3.4)	12.1 (9.1)	3.6(3.6)	12.5 (10.8)	12.7 (11.3)	13.1 (11.8)
**Refinement**							
Resolution (Å)	28.79 - 2.30 (2.38-2.30)	39.12-2.76 (2.85-2.76)	26.45-1.85(1.91-1.85)	41.23-2.59(2.68-2.59)	36.05-1.70(1.76-1.70)	30.11-2.59(2.68-2.59)	29.46-1.40(1.45-1.40)
No. reflections	72208 (4537)	43482 (2548)	35982(3330)	54816(4525)	44367(3286)	13471(1249)	76339（4500）
R_{work} / R_{free}	22.3/26.1	23.2/26.8	19.1/22.9	22.5/24.2	18.6/22.9	19.6/25.3	14.7/17.9
No. atoms							
Protein	12713	12742	3396	12722	3373	3387	3426
Ligand/ion			65		65	72	
Water	625	290	163	322	377	82	469
B-factors							
Protein	35.0	40.9	54.8	45.7	25.2	44.2	24.7
Ligand/ion			67.3		39.6	67.0	
Water	28.9	25.5	44.7	28.5	31.8	37.4	35.0
R.m.s. deviations							
Bond lengths (Å)	0.004	0.004	0.007	0.004	0.007	0.002	0.01
Bond angles (°)	0.61	0.63	0.89	0.60	0.92	0.52	1.53

One crystal was used for each structure.

*Values in parentheses are for highest-resolution shell.

Supplementary Figures

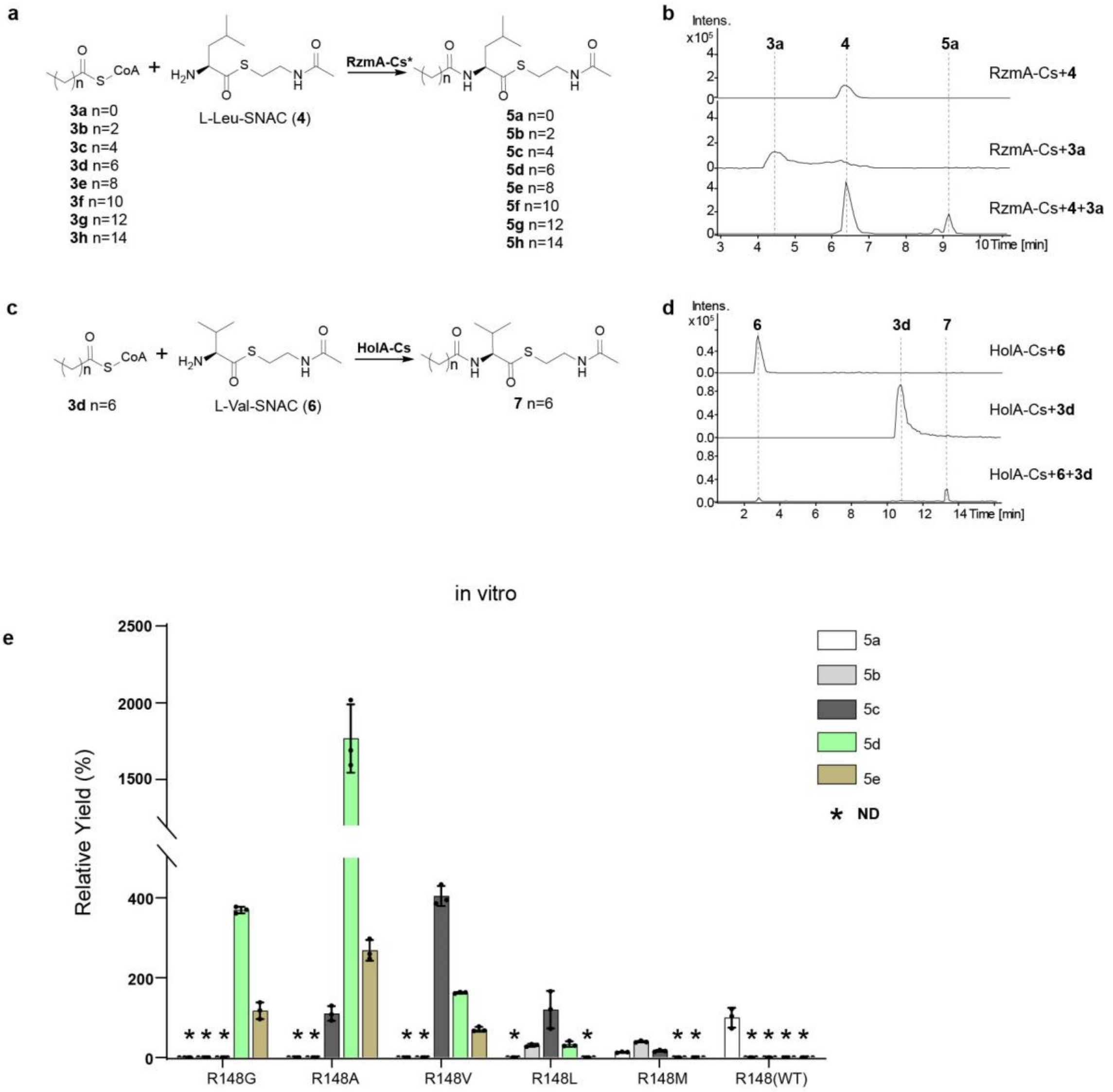

Supplementary Fig. 1. The *in vitro* biochemical experiments of dissected RzmA-Cs and HolA-Cs domains. **a**, The RzmA-Cs catalyzes the condensation between C2-CoA (**3a**) and L-Leu-SNAC (**4**). **b**, UPLC-MS analysis of the product (**5a**) in RzmA-Cs catalyzed reaction. Shown are EICs at m/z = 233.3 ([M+H]$^{+}$, **4**), at m/z = 810.5 ([M+H]$^{+}$, **3a**) and at m/z = 275.3 ([M+H]$^{+}$, **5a**). **c**, The HolA-Cs catalyzes the condensation between C8-CoA (**3d**) and L-Val-SNAC (**6**). **d**, UPLC-MS analysis of the product (**7**) in HolA-Cs catalyzed reaction. Shown are EICs at m/z = 219.3 ([M+H]$^{+}$, **6**), at m/z = 945.8 (**3d**, octanoyl-CoA ammonium salt, CAS Number: 799812-82-1), and at m/z = 345.5 ([M+H]$^{+}$,**7**). **e**, *in vitro* test of RzmA-Cs domain variants. The donor substrates contain same concentrations of C2-, C4-, C6-, and C8-CoAs were reacted with mimic substrates of acceptor L-Leu-SNAC for each Cs domain variants. The yield of **5a** in RzmA-Cs WT was quantified as a reference (100%) for the peak areas of EICs. The products that were not detected (ND) by UPLC-MS are marked with asterisks. Three (n=3) independent tests and data are presented as mean yield ± SD. Source data are provided in the Source Data file.

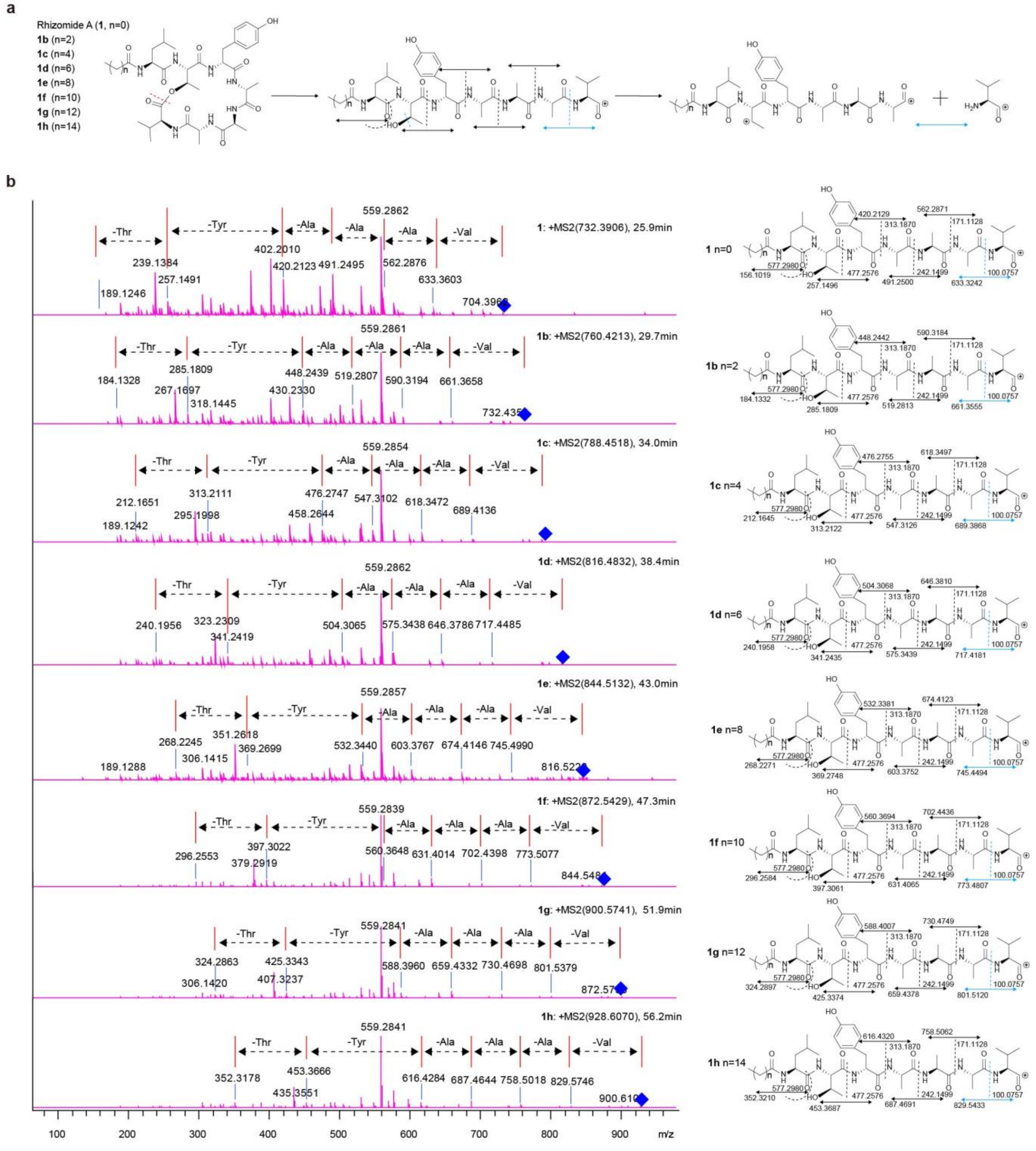

Supplementary Fig. 2. Structural determination of rhizomide derivatives 1b-1h by MS/MS. **a**, Structures and MS/MS fragmentation pattern of rhizomides. **b**, HRMS/MS spectra (left) and theoretical ion peaks of fragments (right) of rhizomide A (**1**) and derivatives **1b**-**1h**. All MS values in the spectra were given automatically from the Compass DataAnalysis software (Bruker).

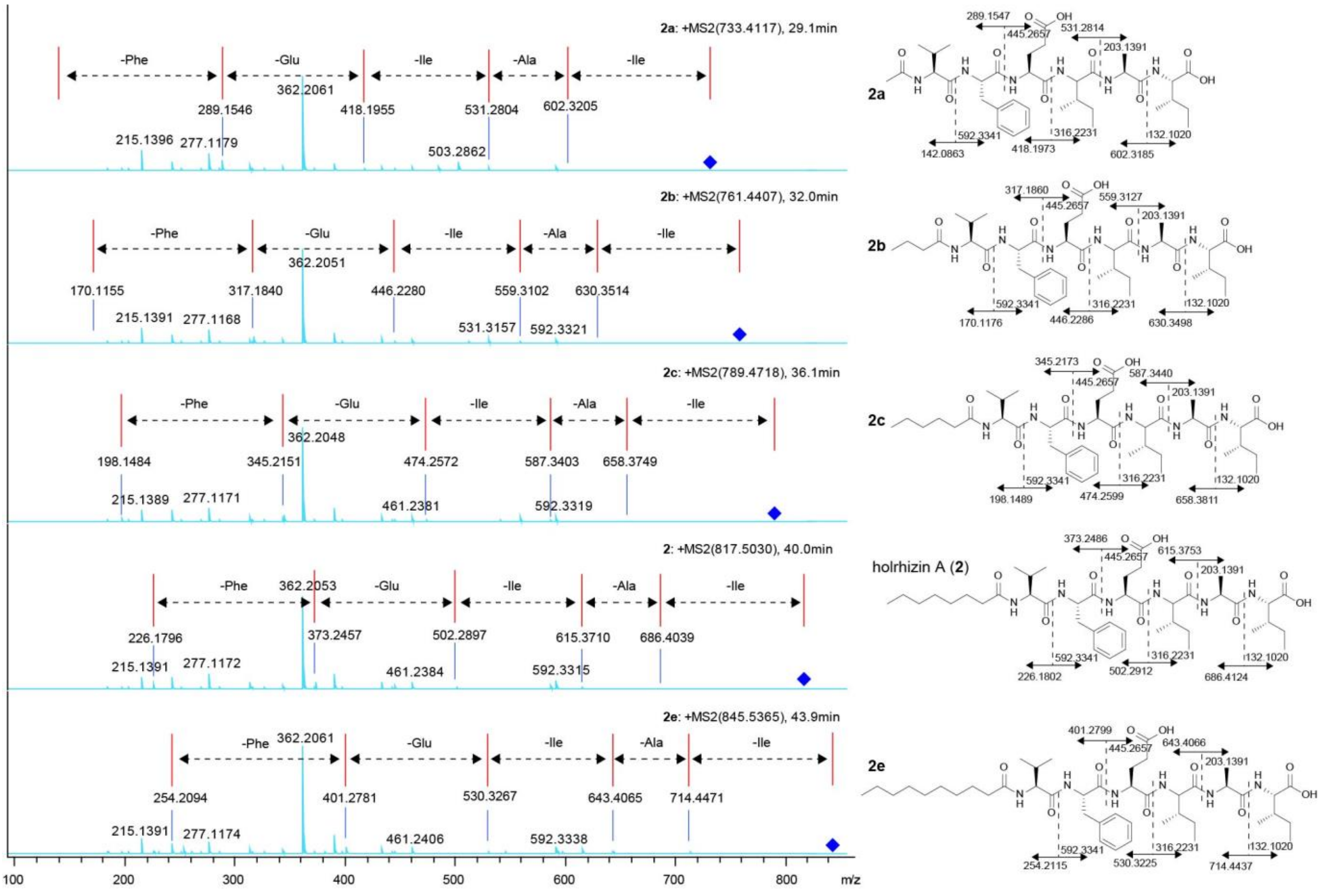

Supplementary Fig. 3. Structural determination of holrhizin derivatives by MS/MS. HRMS/MS spectra (left) and theoretical ion peaks of fragments (right) of holrhizin A (**2**) and derivatives **2a**-**2e**. All MS values in the spectra were given automatically from the Compass DataAnalysis software (Bruker).

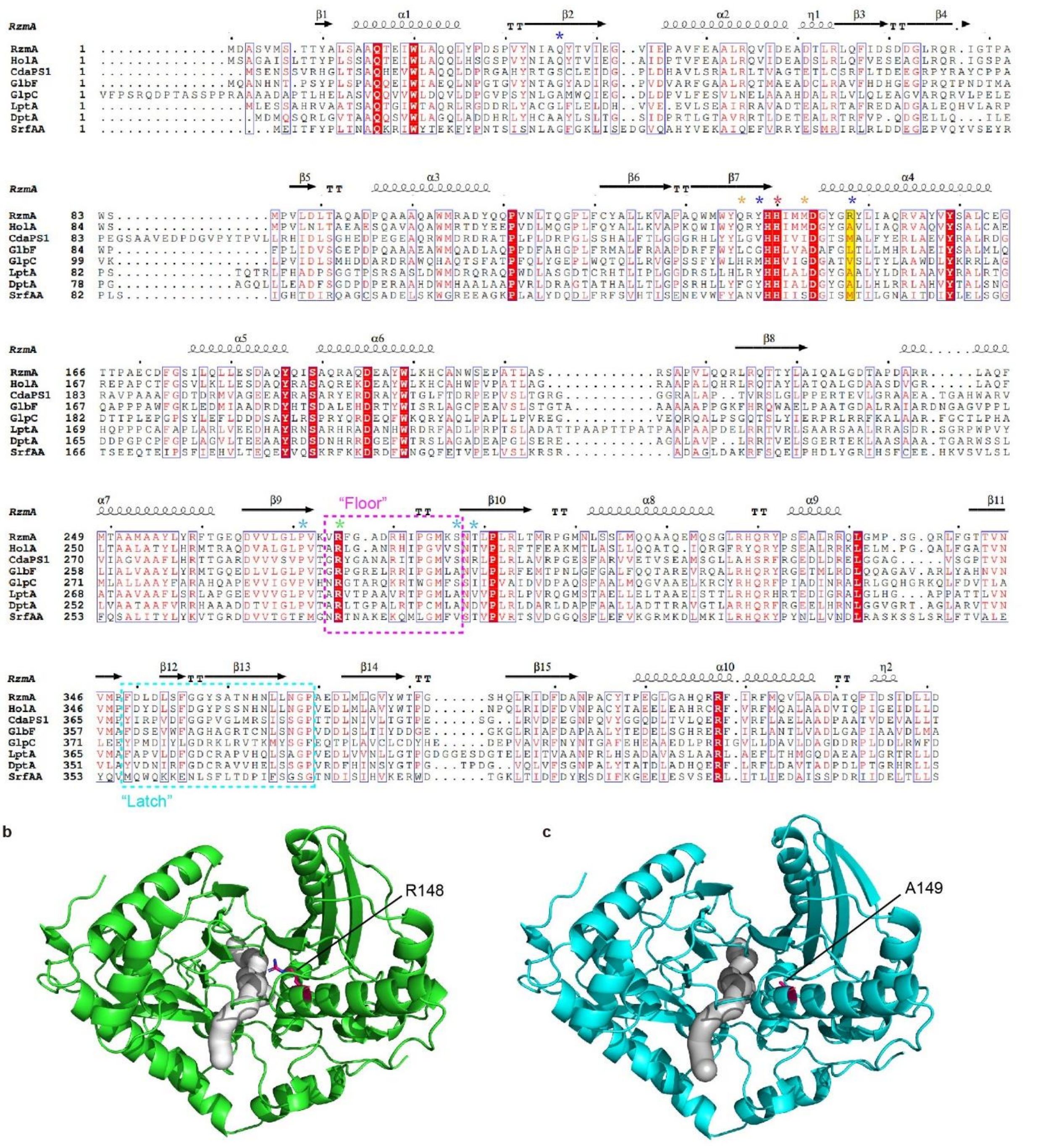

Supplementary Fig. 4. Comparison between RzmA and HolA Cs domains. a, Structure-based sequence alignment of RzmA-Cs and HolA-Cs with other Cs domains (as also shown in Fig. 1d). CdaPS1 (*Streptomyces coelicolor*, PDB code 4JN3), GlbF (*Schlegelella brevitalea*, A8KCJ2), DptA (*Streptomyces filamentosus*, Q50E74), LptA (*Streptomyces fradiae*, Q45R85), SrfAA (*Bacillus subtilis*, P27206). The "floor" and "latch" regions are boxed with magenta and cyan dashed lines, respectively. The blue asterisks (Q36, Y138 and R148 of RzmA) and yellow asterisks (M143 and Q136 of RzmA) represent key and additional sites for specificity of lipid chains, respectively. The active site residue H140 of RzmA is marked as red. Three potential residues (P271, S287 and T289 of RzmA) for specificity of amino acid substrate are labelled in cyan. The green asterisk residue (R275) within the "floor" loop is critical for the conformational stability during substrate binding. **b** and **c**, The predicted structures of RzmA-Cs and HolA-Cs by homology modeling, with their active-site tunnels and the "gate" residues (R148 and A149) highlighted respectively.

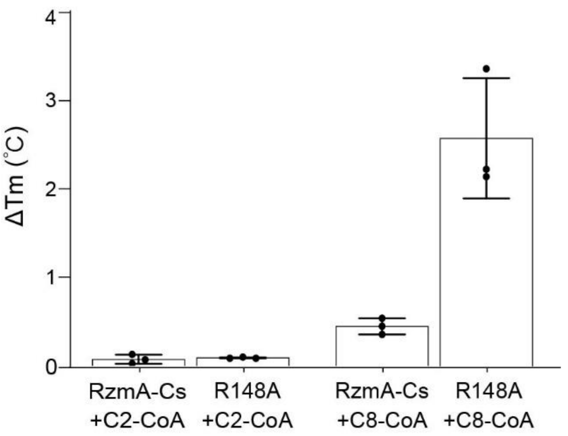

Supplementary Fig. 5. Substrate selection of the RzmA-Cs mutants. Binding of RzmA-Cs wild-type and its mutant R148A to the substrates C2-CoA or C8-CoA, as measured by the protein thermal shift assays. Test were conducted in triplicates (n=3, and mean yield ± SD are presented). Source data are provided in the Source Data file.

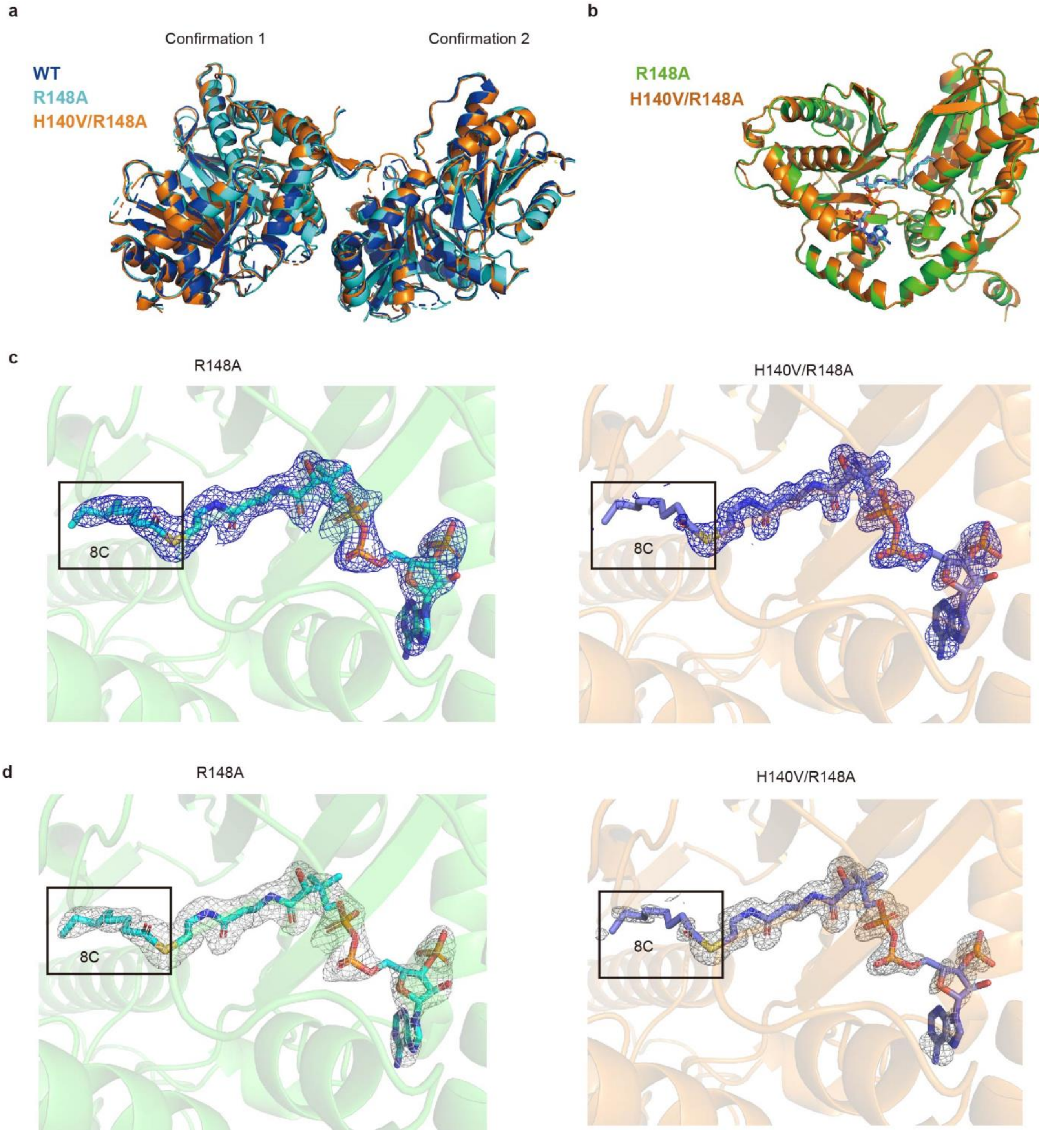

Supplementary Fig. 6. Crystal structures of RzmA-Cs mutants in complex with the C8-CoA substrate. a, Superimposition of three unbound structures of RzmA-Cs. **b**, Comparison of C8-CoA-bound structures between RzmA-Cs R148A and H140V/R148A. **c**, The 2Fo-Fc density map of C8-CoA in R148A (cyan) and H140V/R148A (purple), with contour level at 1 σ and shown as blue mesh. **d**, The Fo-Fc omit map of C8-CoA in R148A (cyan) and H140V/R148A (purple), with contour level at 3.0 and shown as gray mesh.

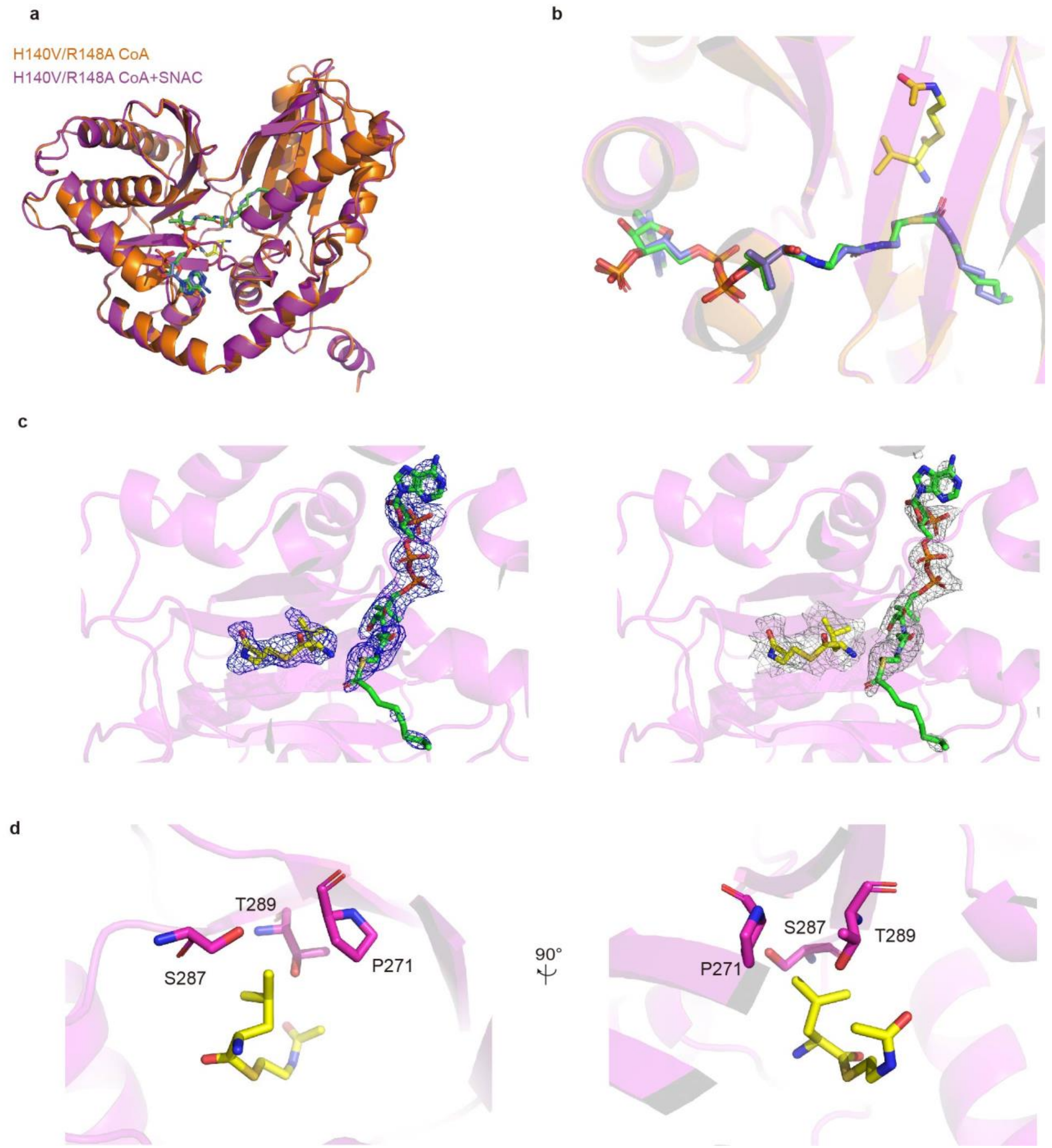

Supplementary Fig. 7. Crystal structure of RzmA-Cs mutant H140V/R148A in complex with two substrates. **a**, Superimposition of H140V/R148A structure in co-complex with C8-CoA (green), L-Leu-SNAC (yellow) and that with only C8-CoA (purple). **b**, An enlarged view of the substrate-binding pocket. **c**, The 2Fo-Fc density map of C8-CoA and L-Leu-SNAC (left, contour level = 0.8 σ) and the Fo-Fc omit map of C8-CoA and L-Leu-SNAC (right, contour level = 3.0 σ). **d**, Residues close to the side-chain of substrate L-Leu in RzmA-Cs, shown in two views.

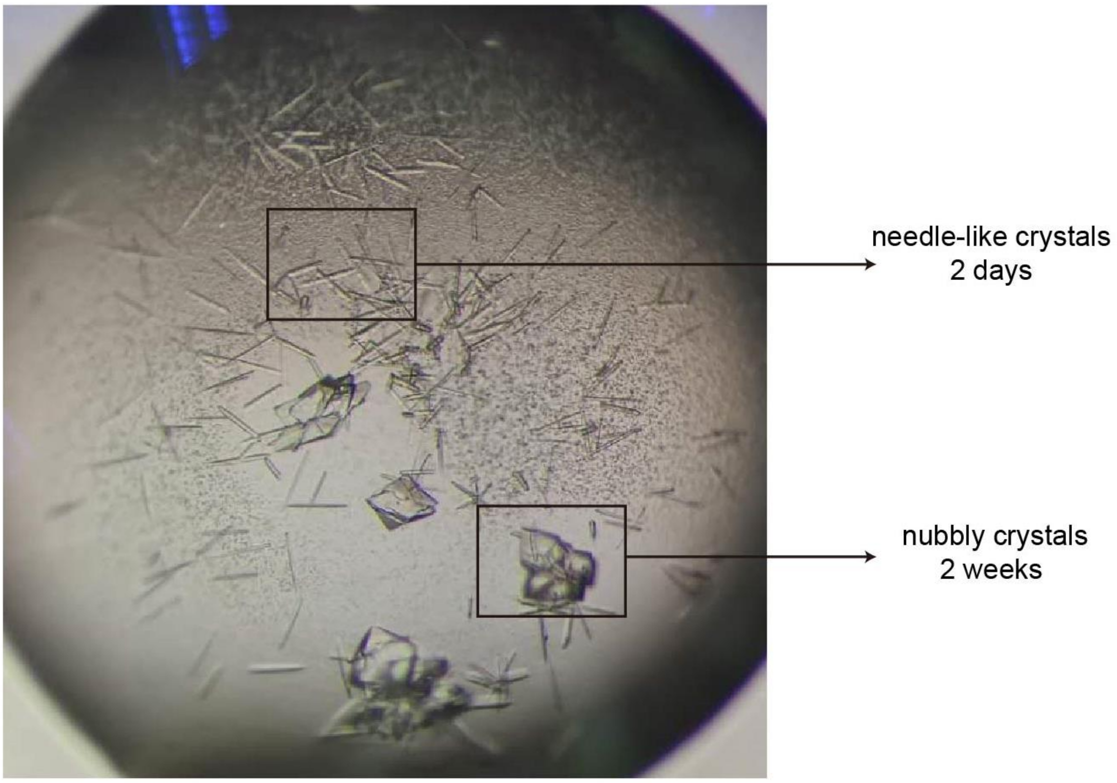

Supplementary Fig. 8. A picture showing two types of RzmA-Cs H140V/R148A crystals growing in the same drop. The needle-like crystals were co-complex with two substrates which grew in two days, and the nubbly crystals grew in the same drop after 2 weeks that were ligand-free state we named “released”.

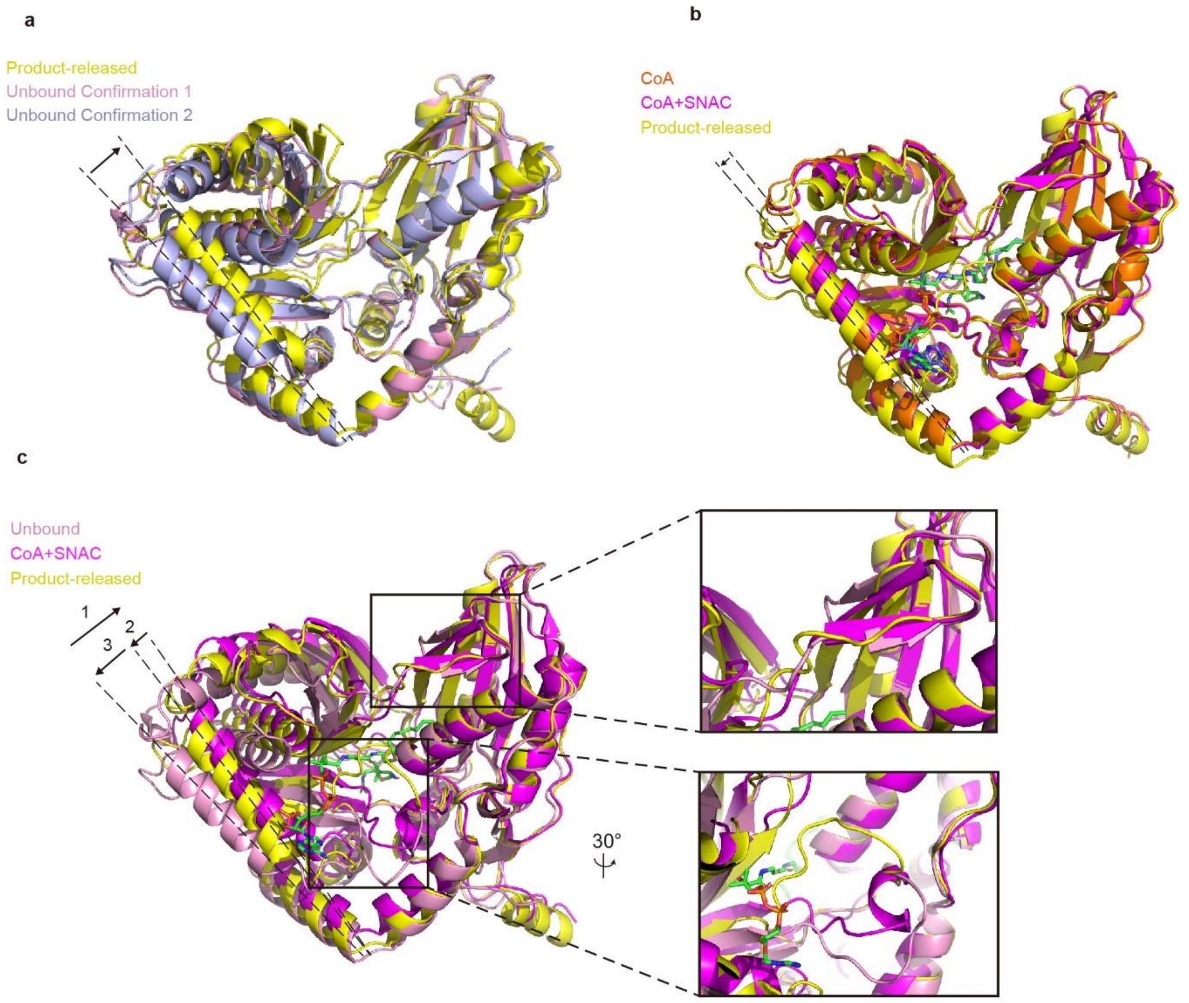

Supplementary Fig. 9. Structure comparison of RzmA-Cs mutant H140V/R148A in different forms. a, Superimposition of H140V/R148A "product-released" structure (yellow) with its two other conformations in the "unbound" form. **b**, Superimposition of H140V/R148A "product-released" structure (yellow) with its co-crystal structures in complex with only one (orange) or two (magenta) substrates. **c**, Superimposition of H140V/R148A structures in the proposed "unbound-bound-released" reaction cycle (illustrated as 1, 2 and 3 in the figure), with two enlarged views showing details at the "latch" and "floor" regions.

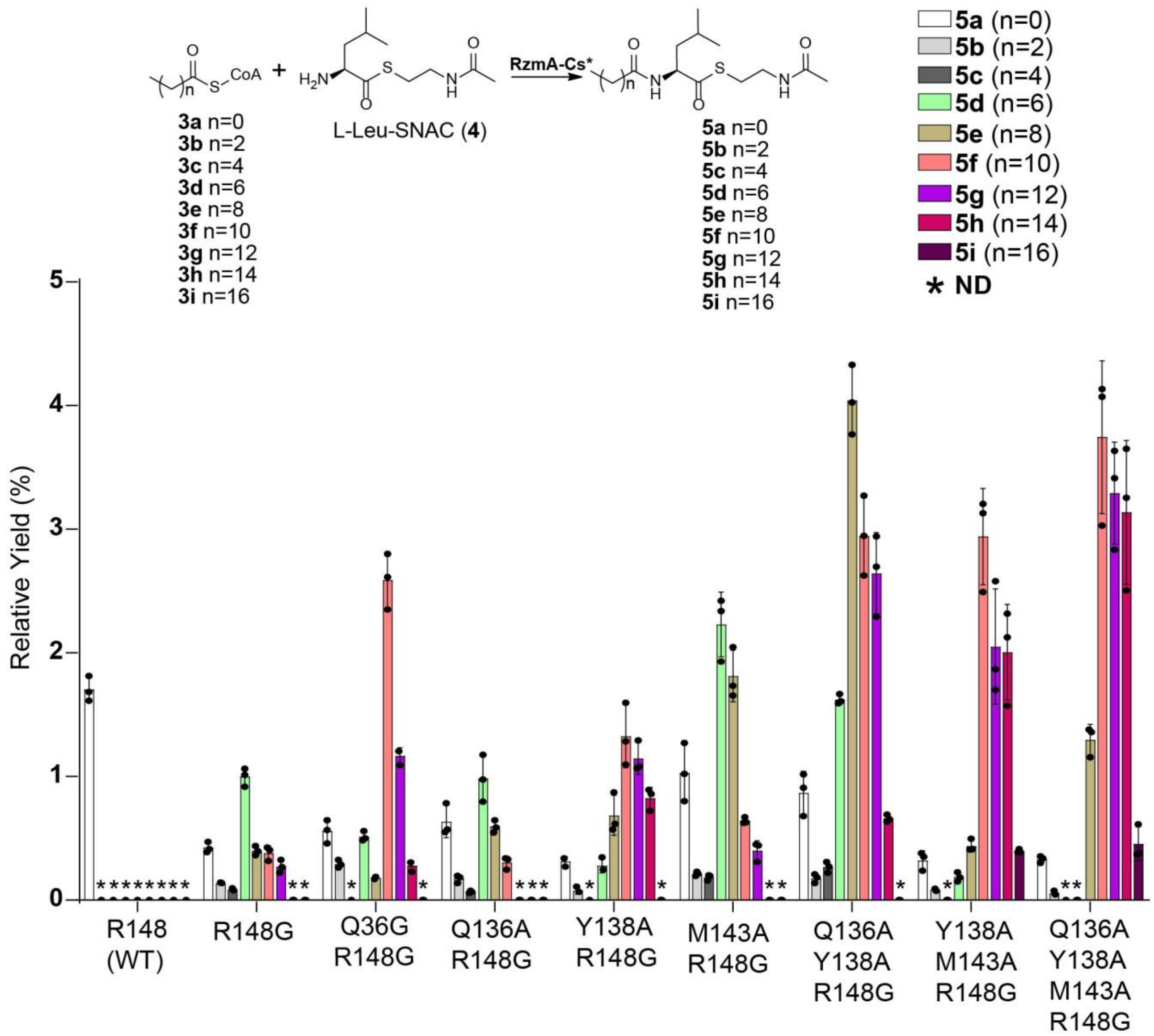

Supplementary Fig. 10 The *in vitro* biochemical experiments of dissected RzmA-Cs to simulate *in vivo* conditions. The relative concentrations of C2-CoA: C4-CoA: C6-C18 CoA (1000:100:1) and an extended incubation time (6 h) were given to simulate in *vivo* condition as much as possible. The relative yields of acyl-Leu-SNAC (**5a-5i)** in the *in vitro* acyl-CoAs substrate competition experiment of RzmA-Cs variants, and the yield of **5d** (C8-Leu-SNAC) in RzmA-Cs R148G was quantified as a reference (100%) for the peak area of EICs. The products that were not detected (ND) by UPLC-MS are marked with asterisks. Three (n=3) independent experiments, data, and mean yield ± SD are presented. Source data are provided as in the Source Data file.

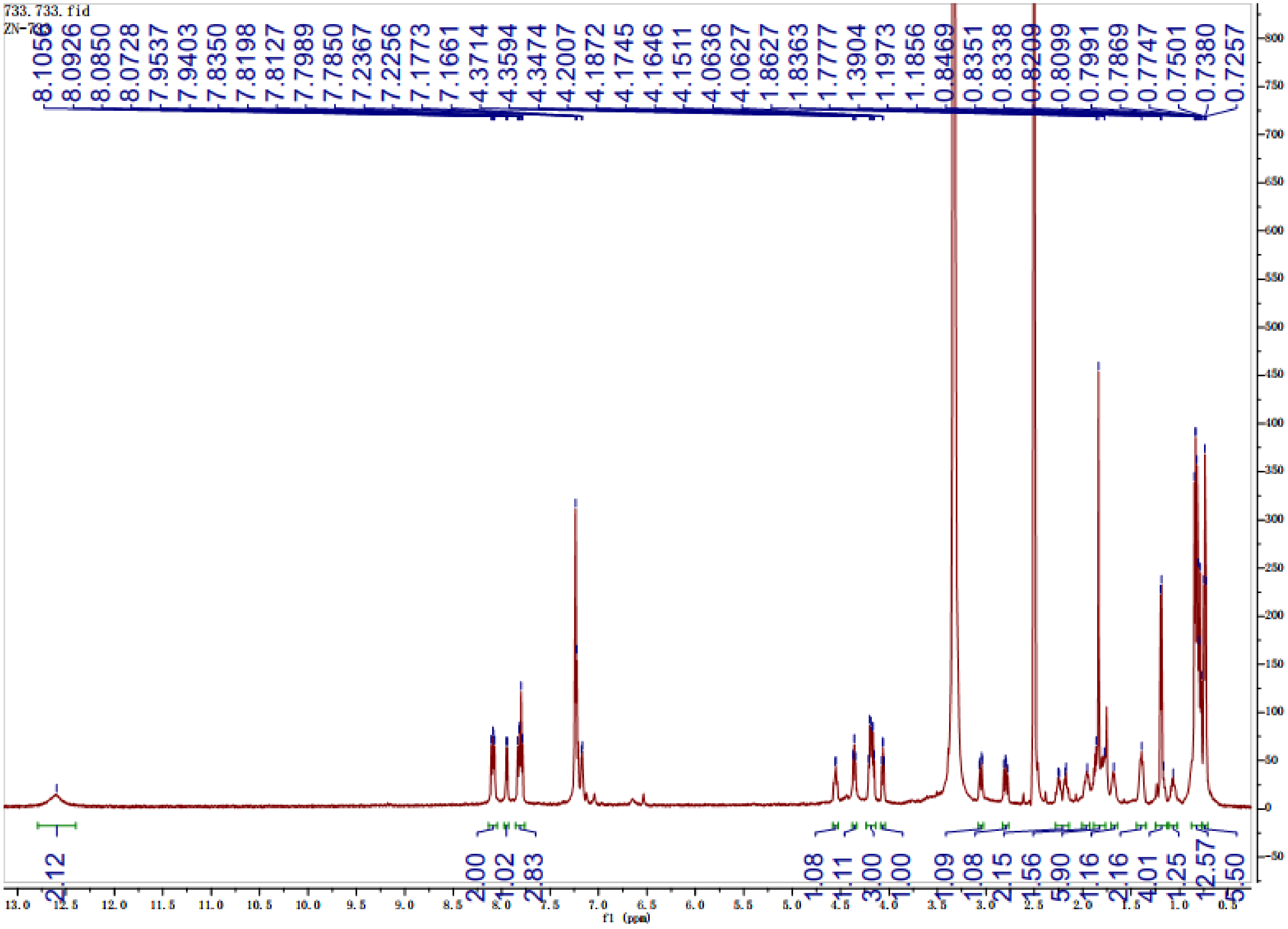

Supplementary Fig. 11. ^{1}H NMR spectrum of **2a** in DMSO-*d*6

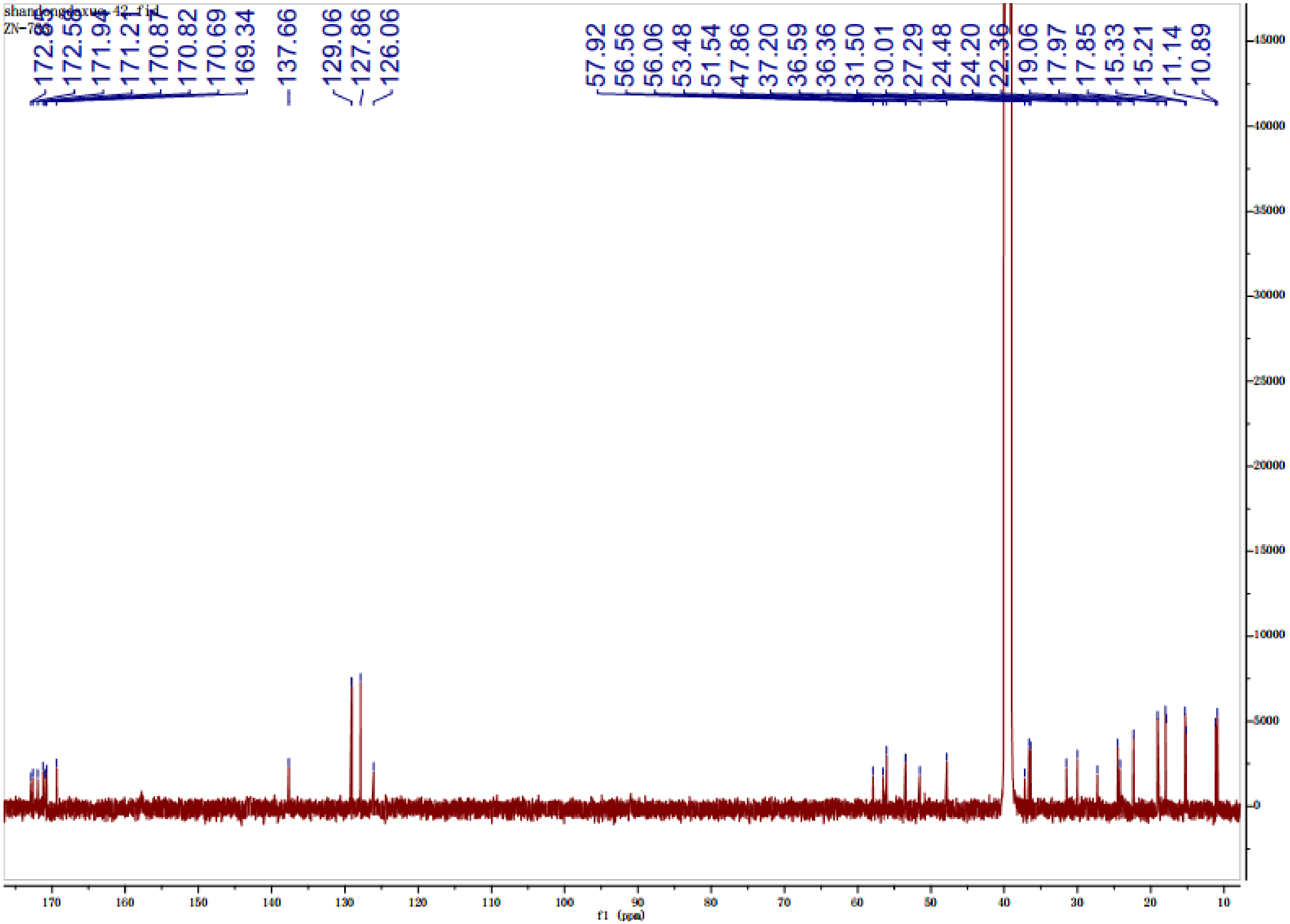

Supplementary Fig. 12. ^{13}C NMR spectrum of **2a** in DMSO-d_6

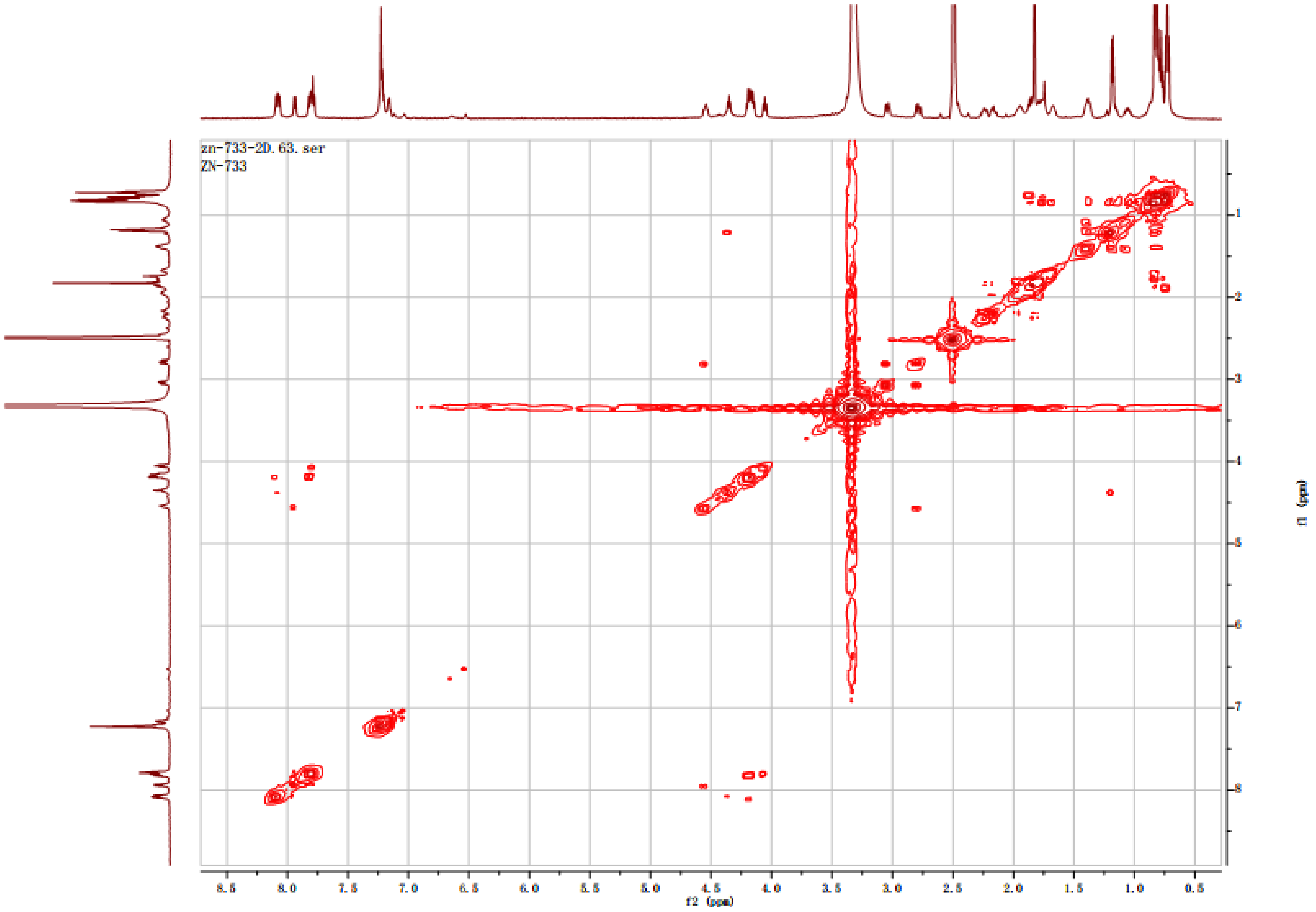

Supplementary Fig. 13. ^{1}H-^{1}H COSY spectrum of **2a** in DMSO-*d*6

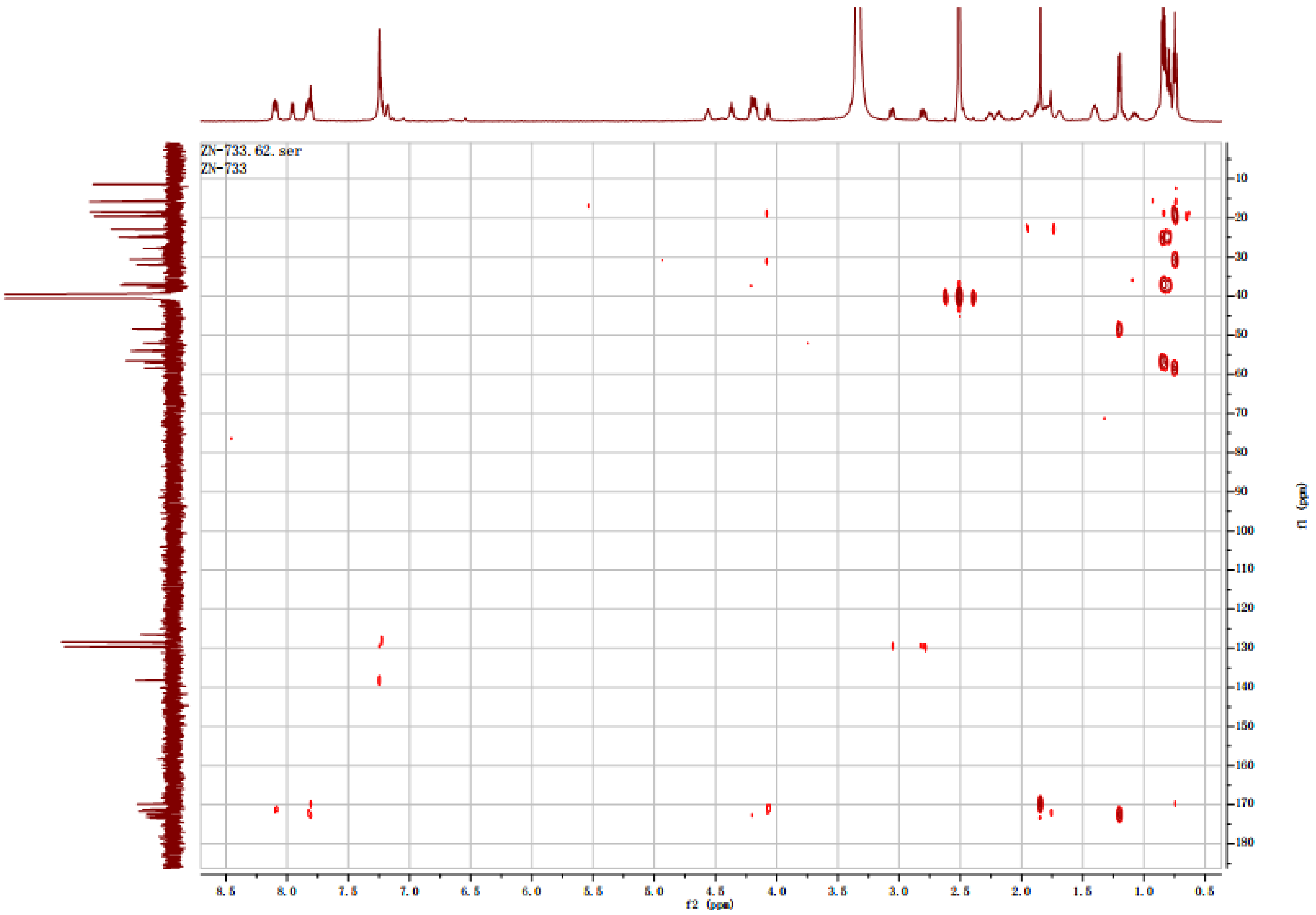

Supplementary Fig. 14. HMBC spectrum of **2a** in DMSO-d_6

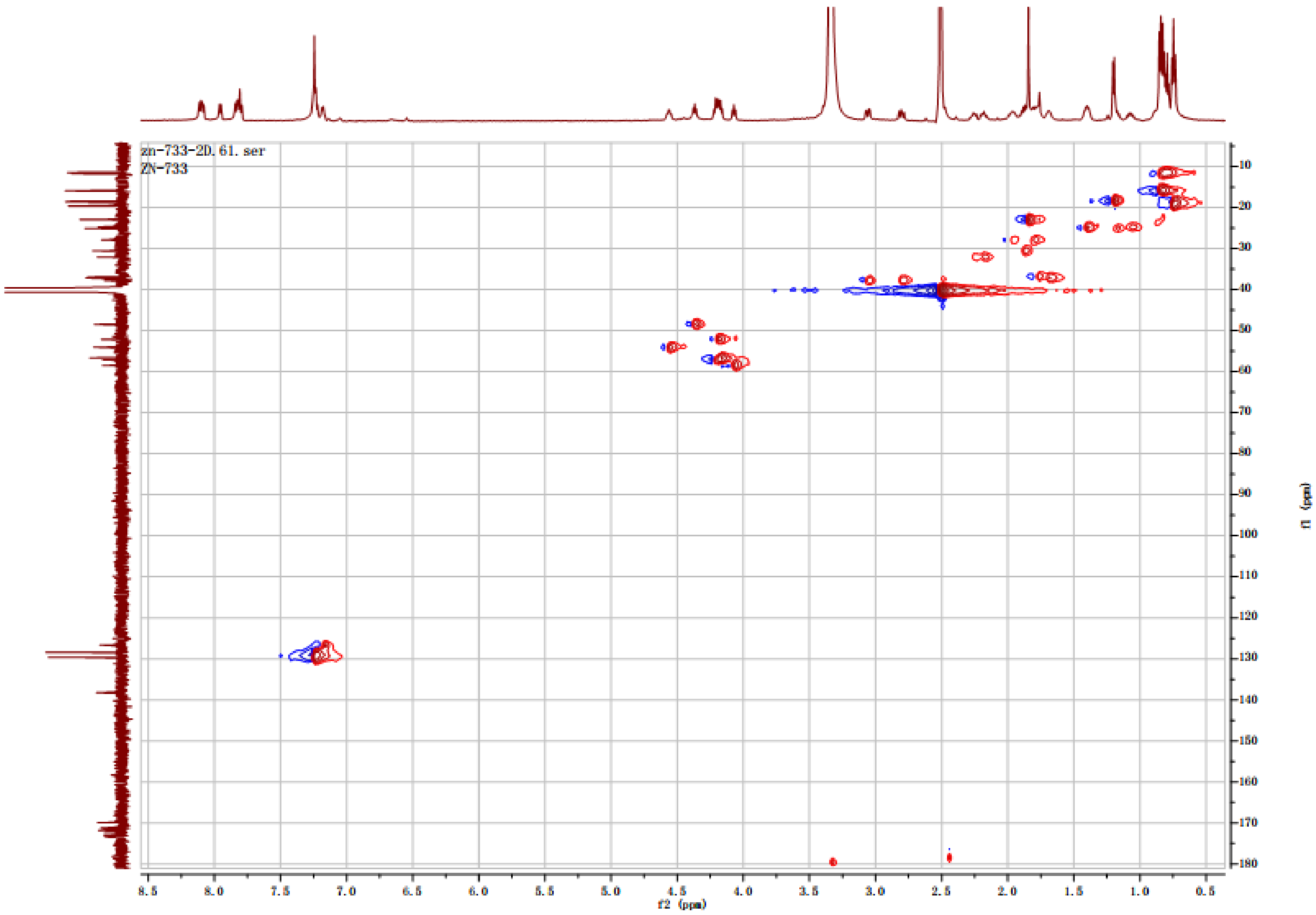

Supplementary Fig. 15. HSQC spectrum of **2a** in DMSO-d_6

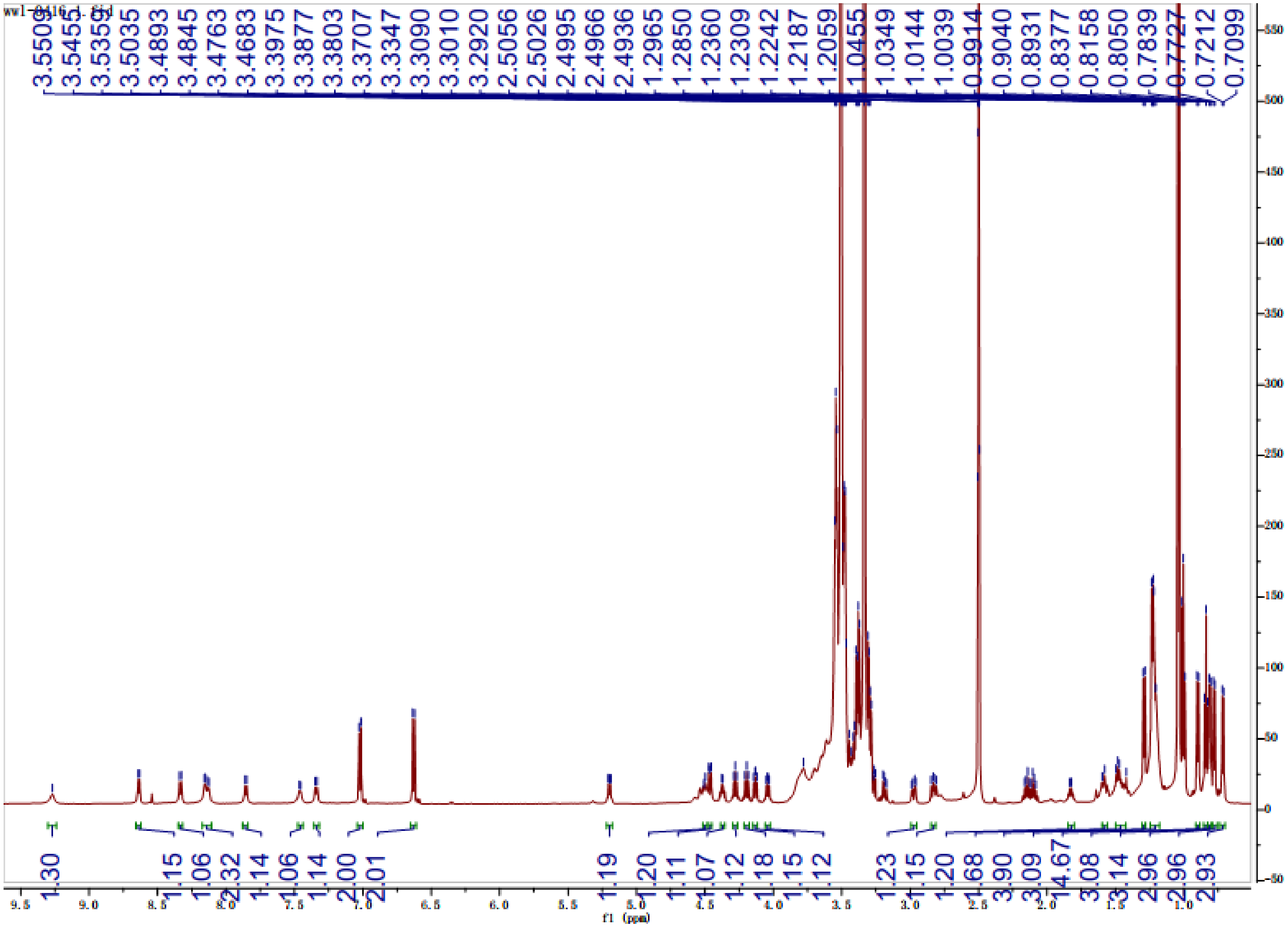

Supplementary Fig. 16. ^{1}H NMR spectrum of **1d** in DMSO-d_6

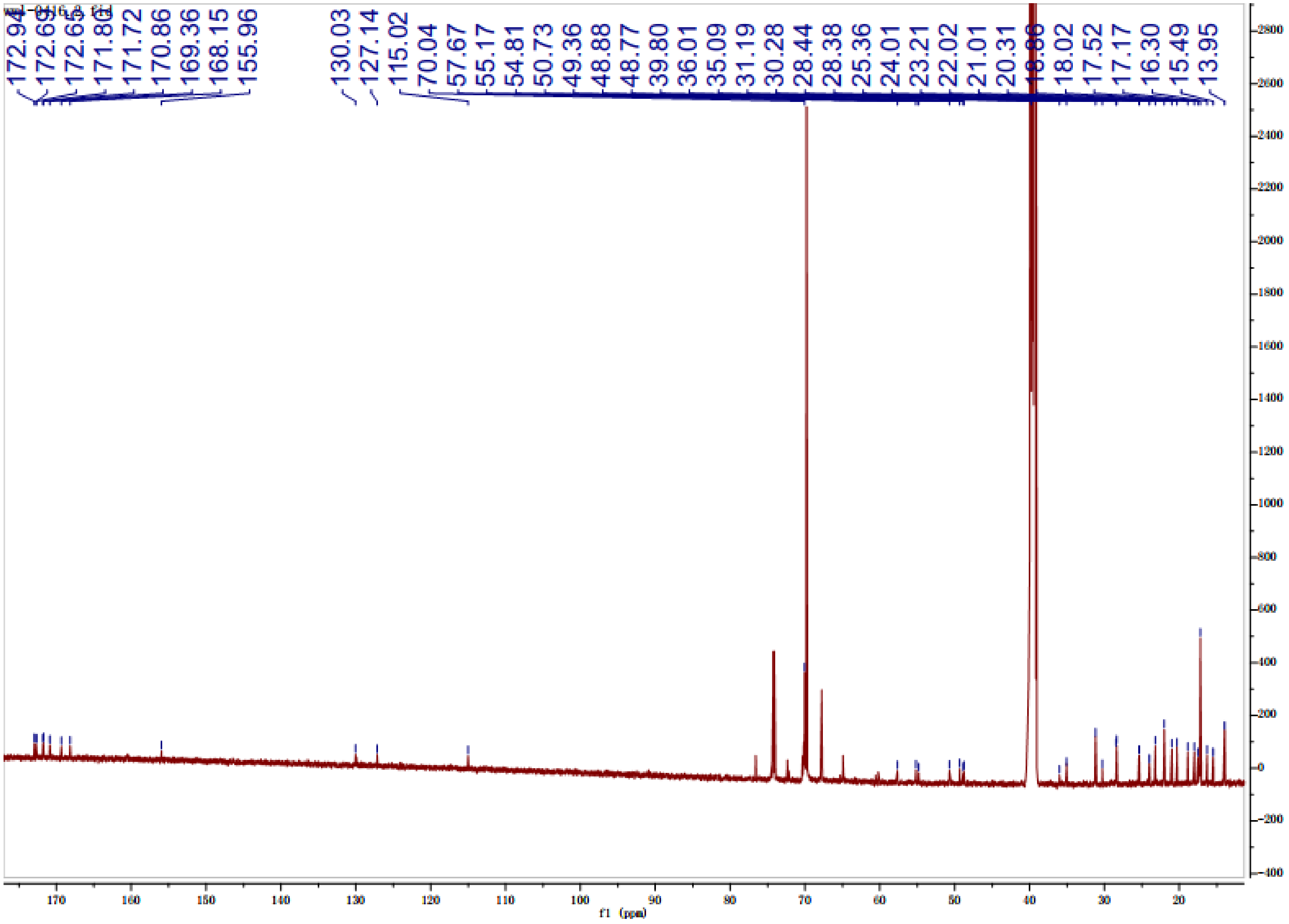

Supplementary Fig. 17. ^{13}C NMR spectrum of **1d** in DMSO-d_6

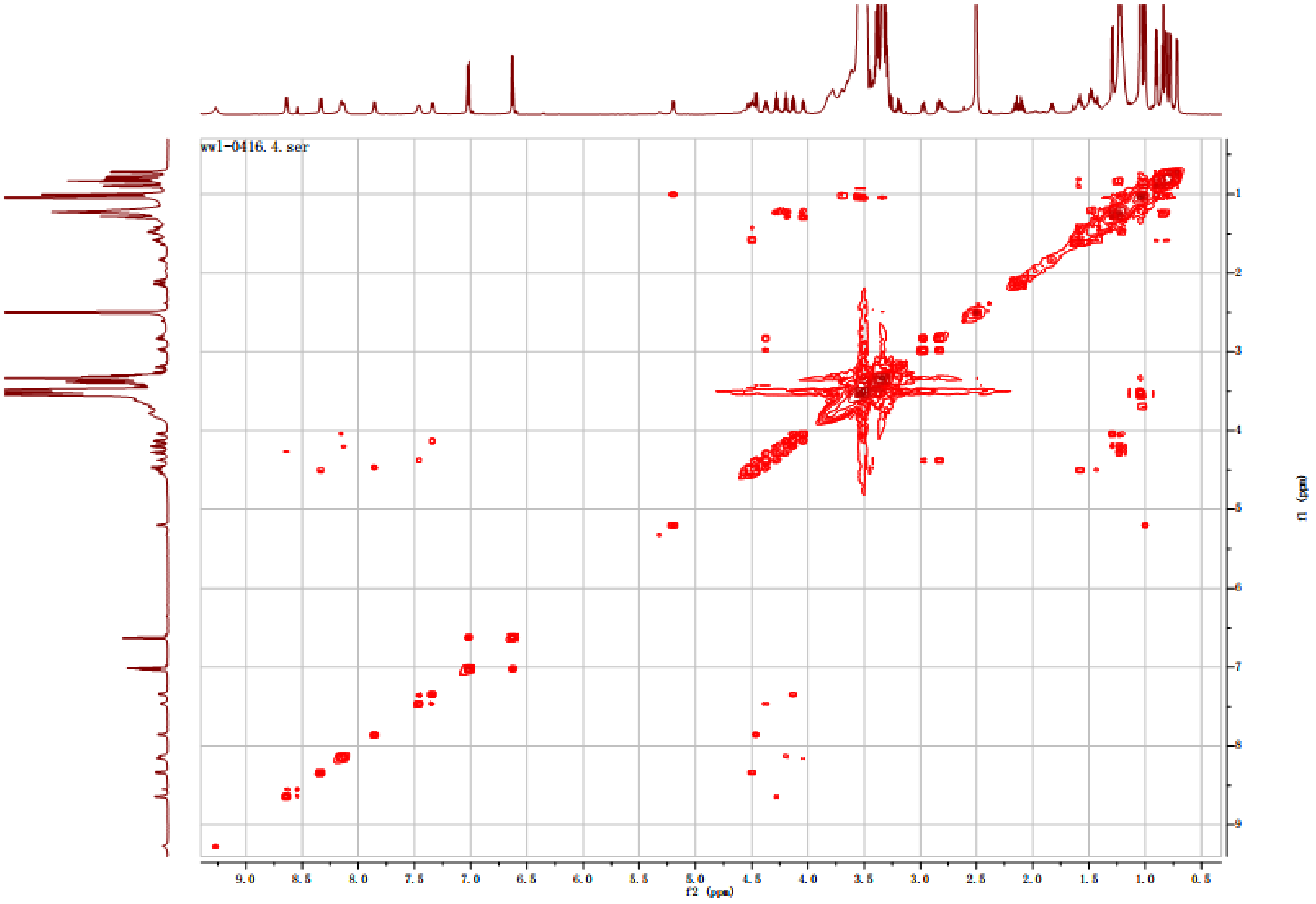

Supplementary Fig. 18. ^1H-^1H COSY spectrum of **1d** in DMSO-d_6

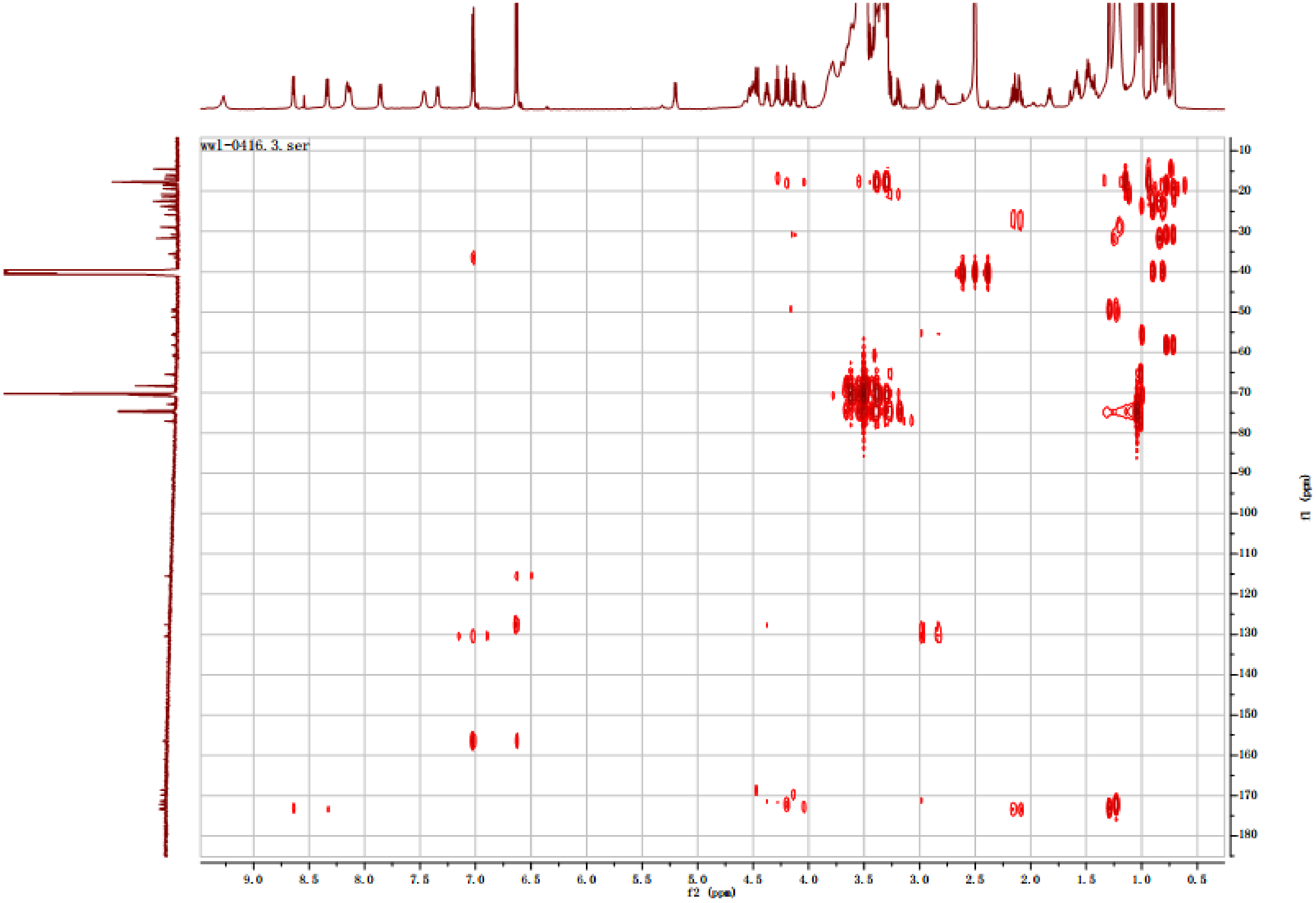

Supplementary Fig. 19. HMBC spectrum of **1d** in DMSO-d_6

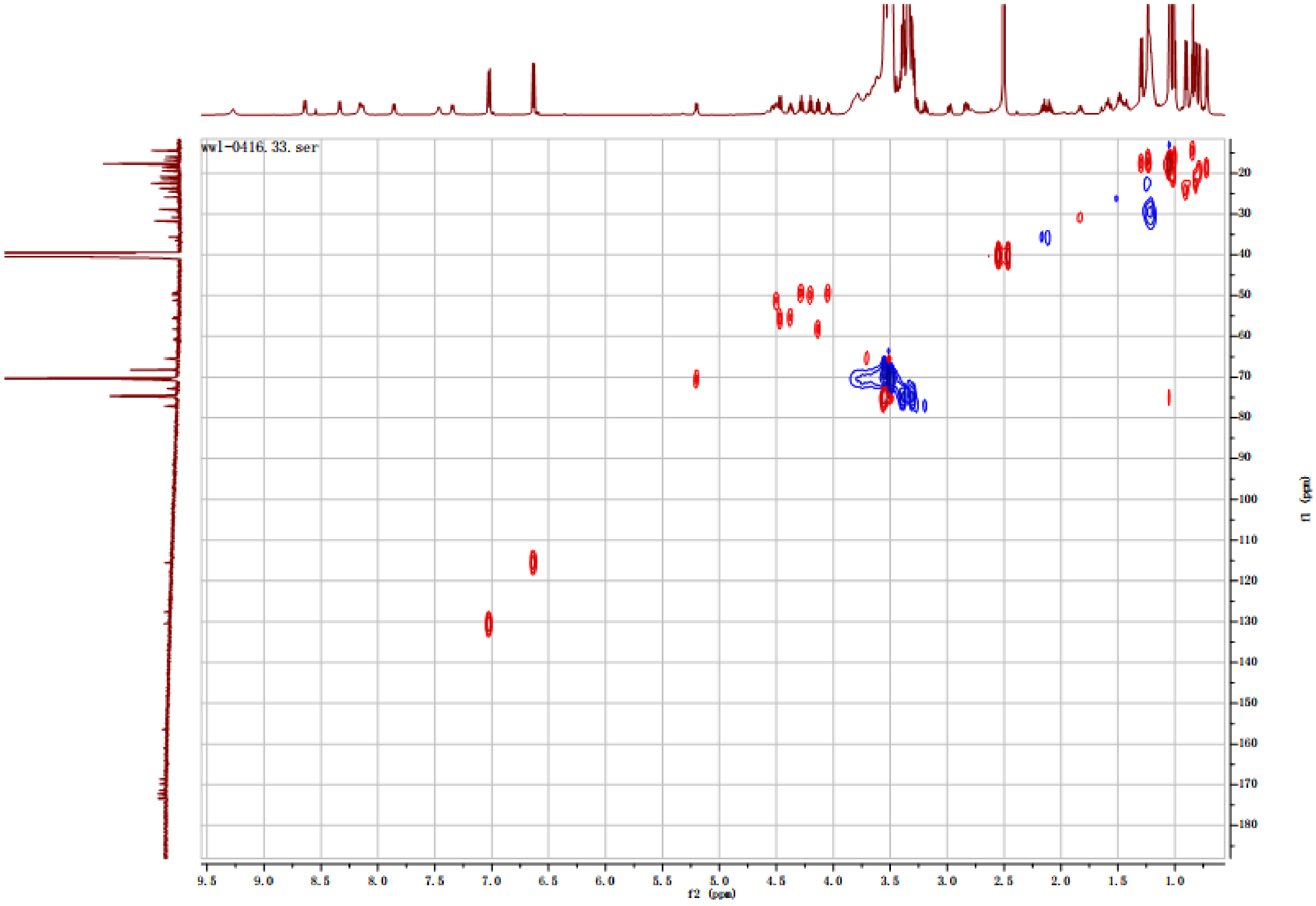

Supplementary Fig. 20. HSQC spectrum of **1d** in DMSO-*d*6

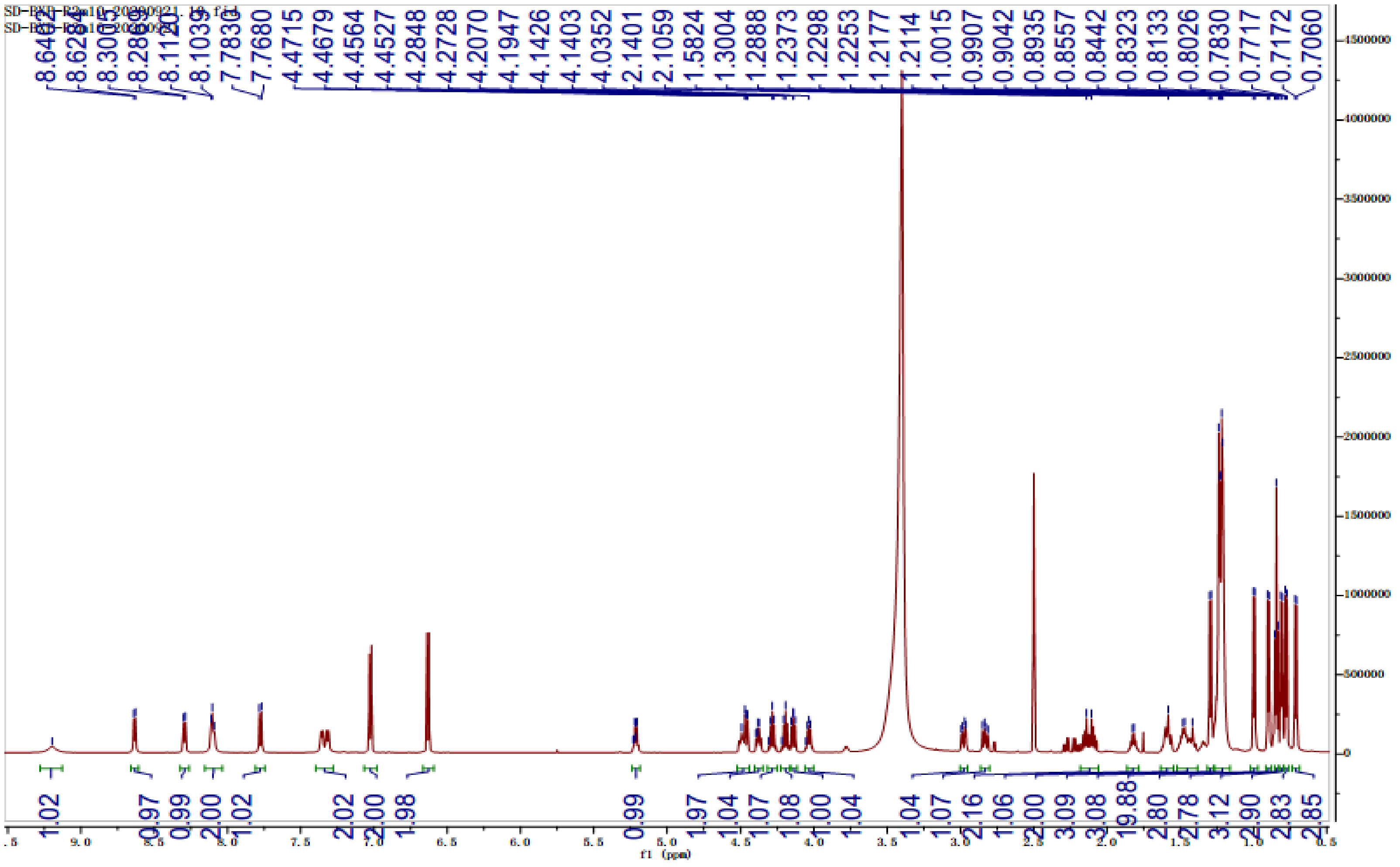

Supplementary Fig. 21. ^{1}H NMR spectrum of **1e** in DMSO-*d*6

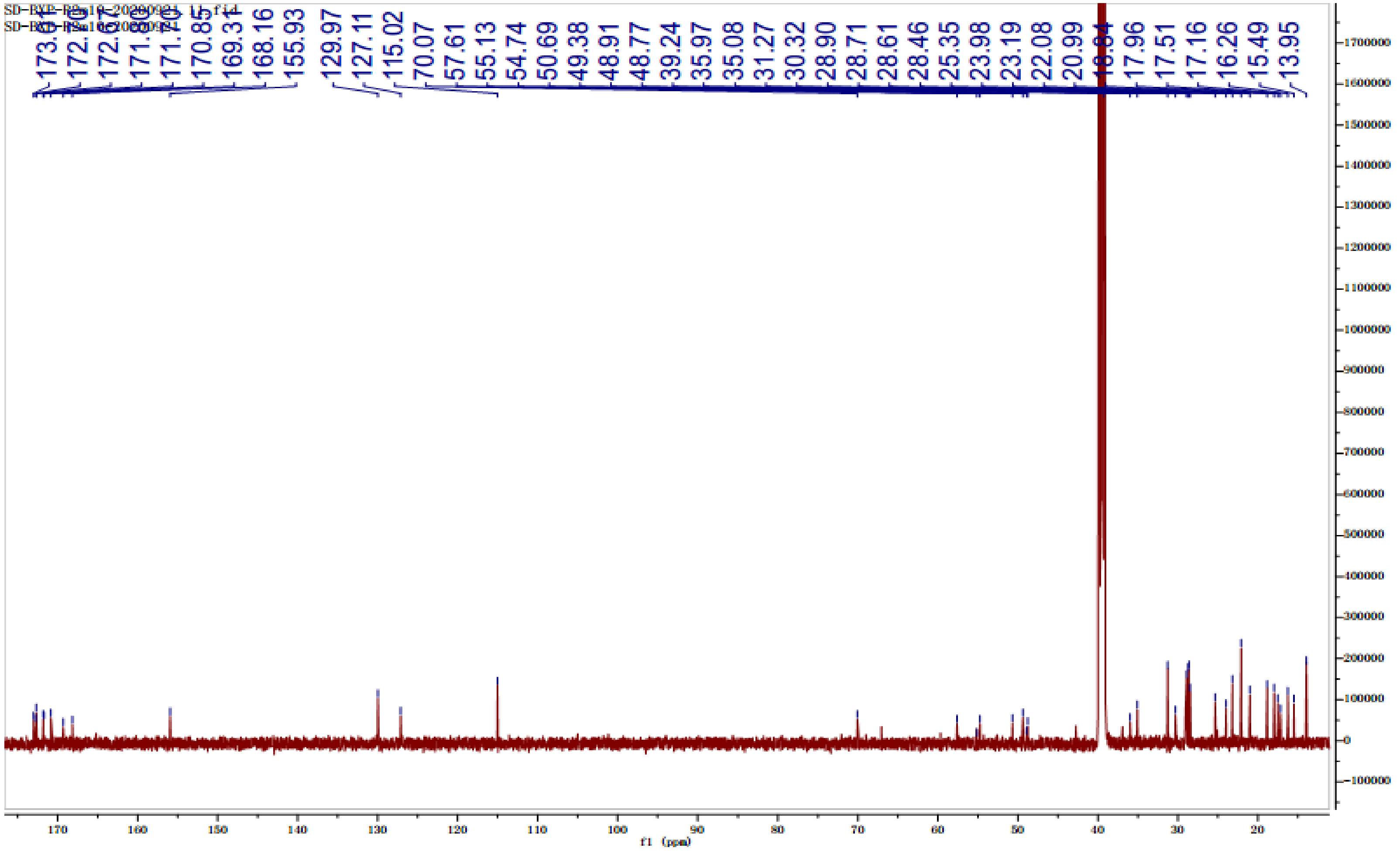

Supplementary Fig. 22. ^{13}C NMR spectrum of **1e** in DMSO-d_6

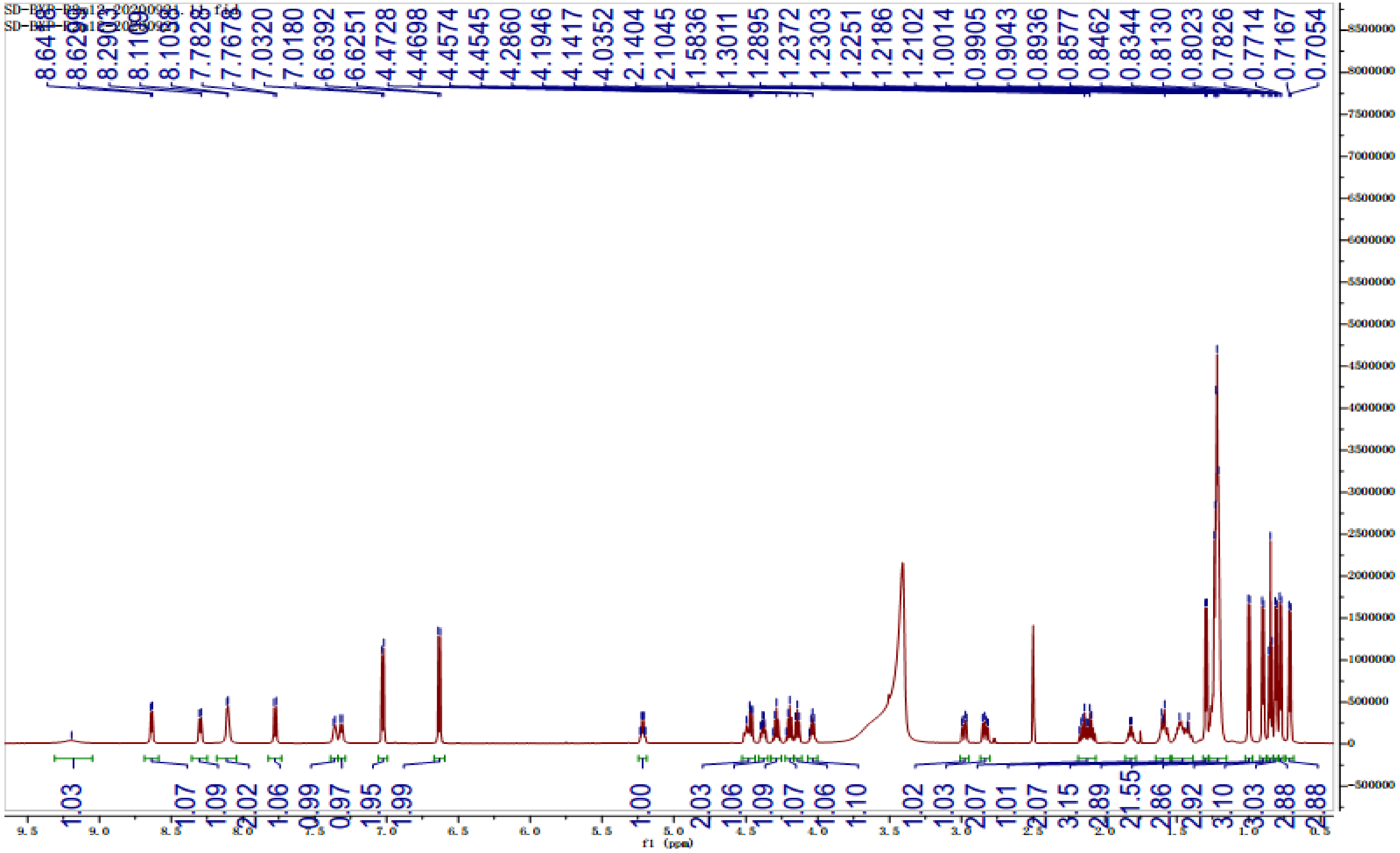

Supplementary Fig. 23. ^{1}H NMR spectrum of **1f** in DMSO-d_6

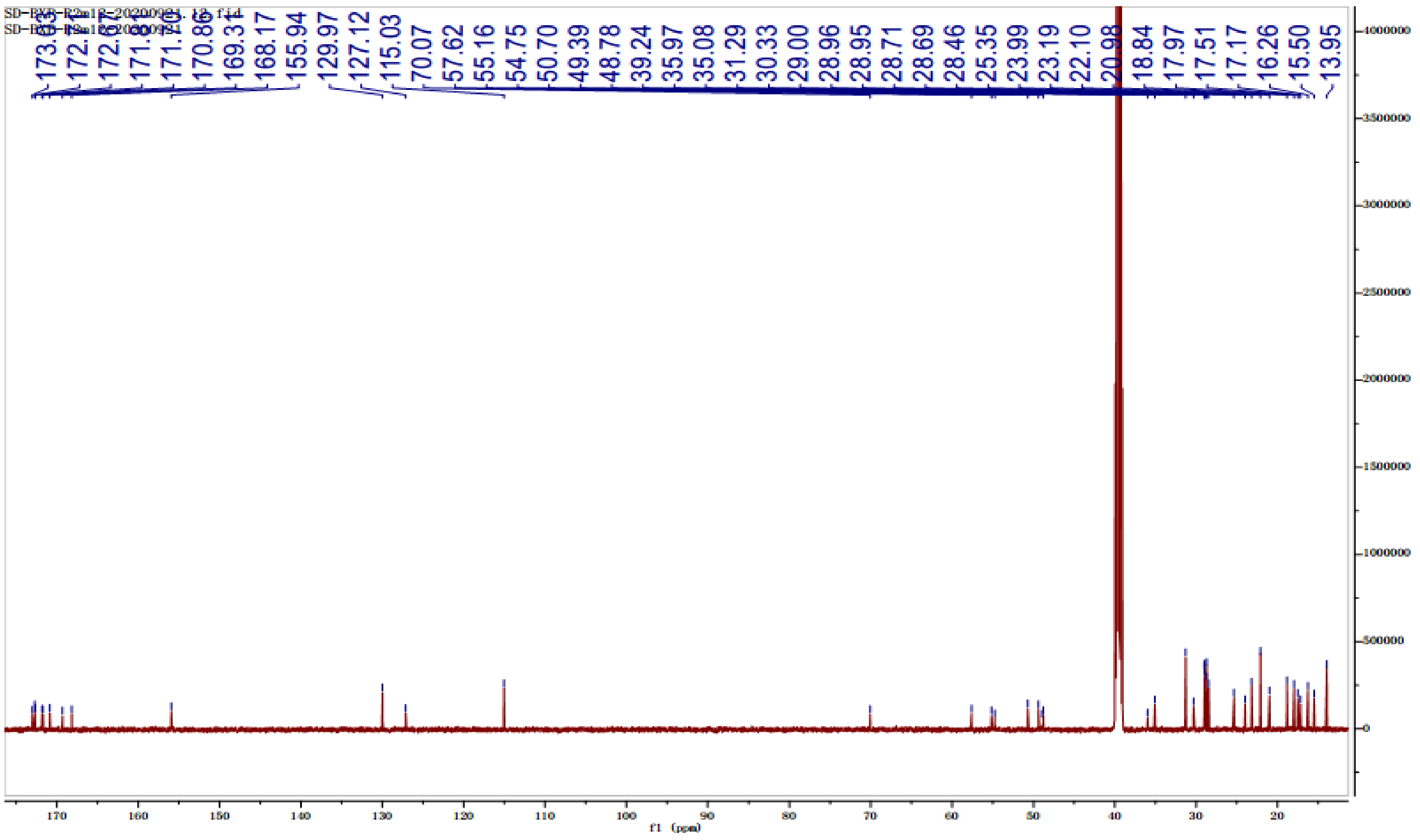

Supplementary Fig. 24. ^{13}C NMR spectrum of **1f** in DMSO-d_6

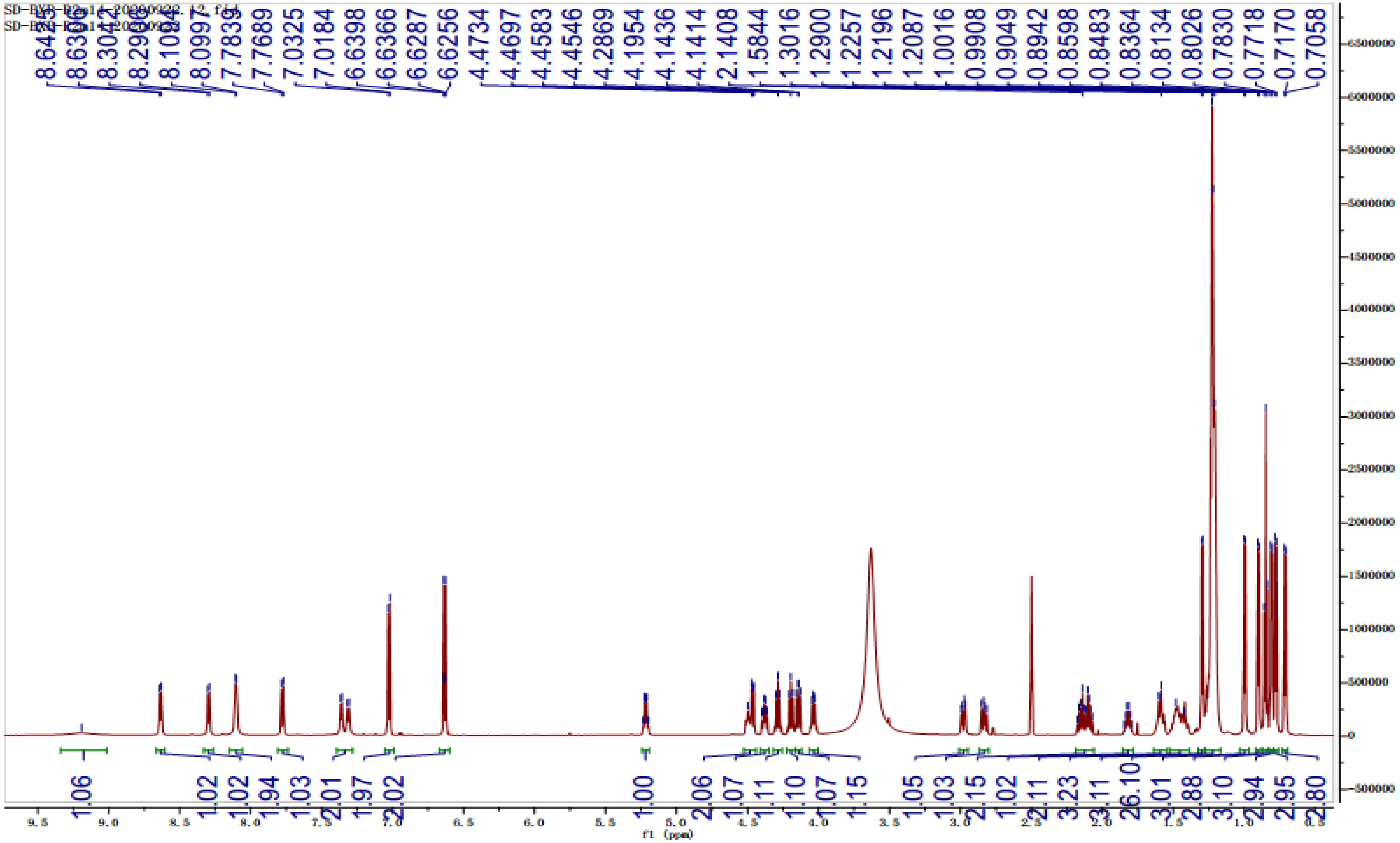

Supplementary Fig. 25. ^{1}H NMR spectrum of **1g** in DMSO-d_6

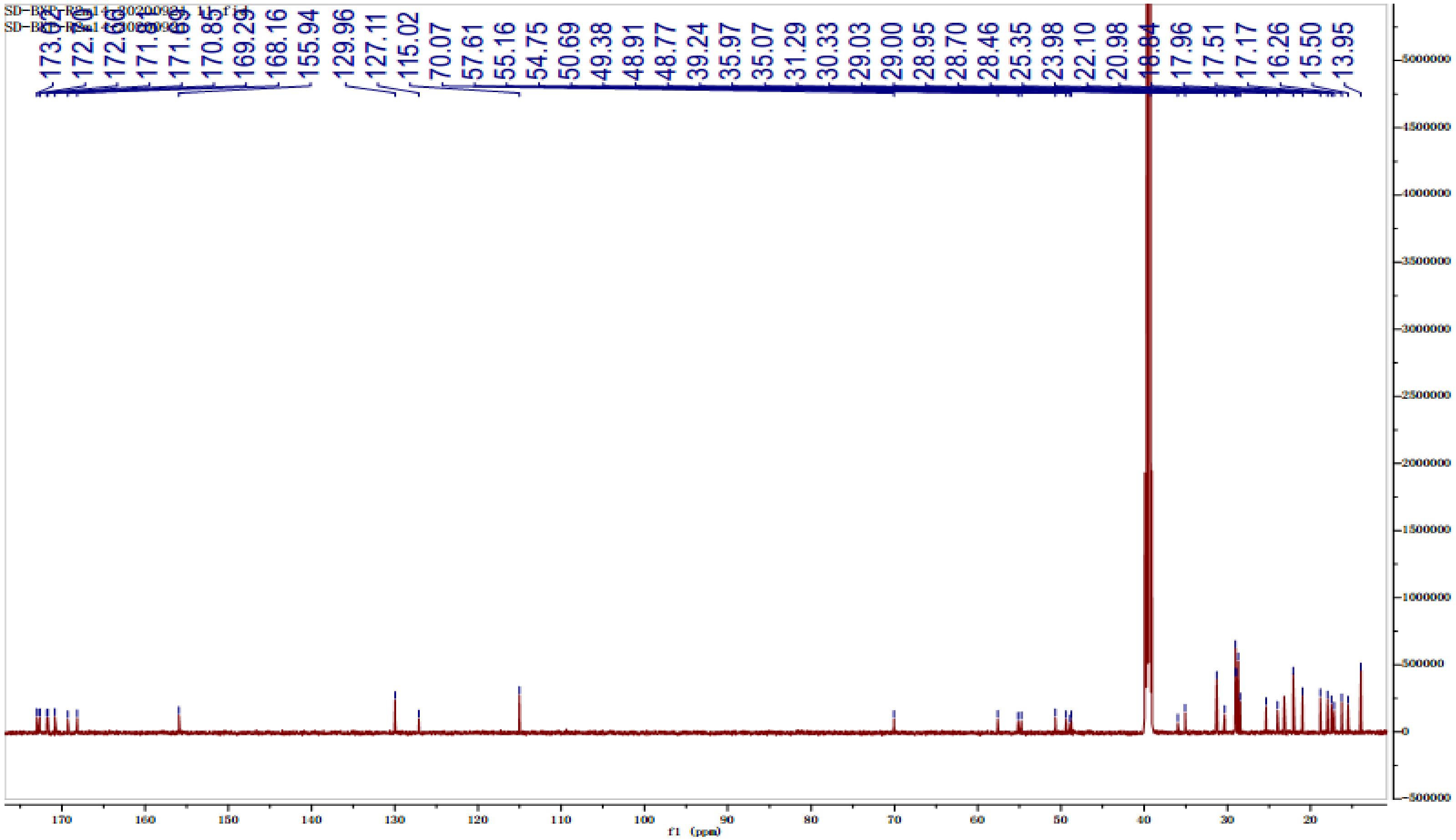

Supplementary Fig. 26. ^{13}C NMR spectrum of **1g** in DMSO-d_6

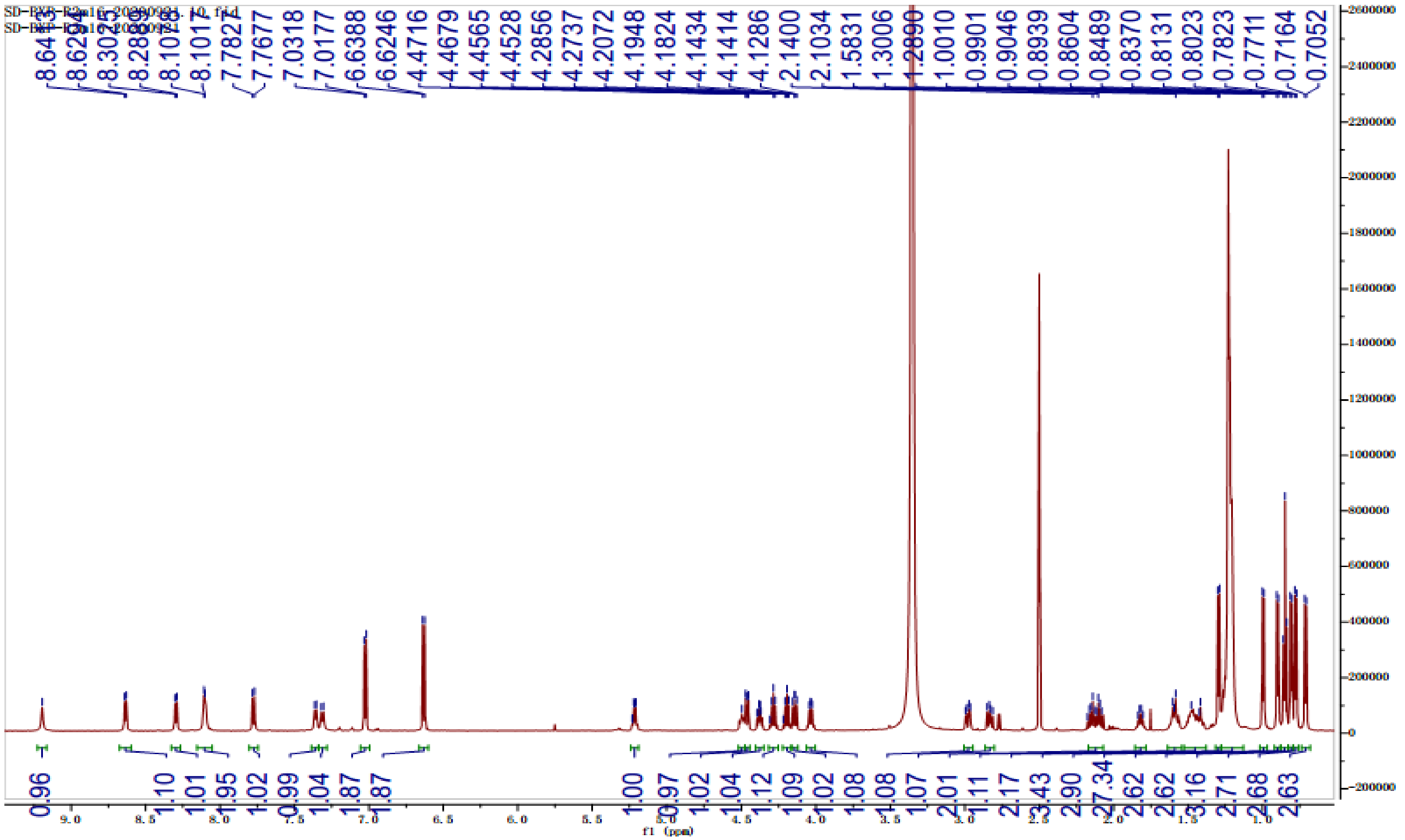

Supplementary Fig. 27. ^{1}H NMR spectrum of **1h** in DMSO-d_6

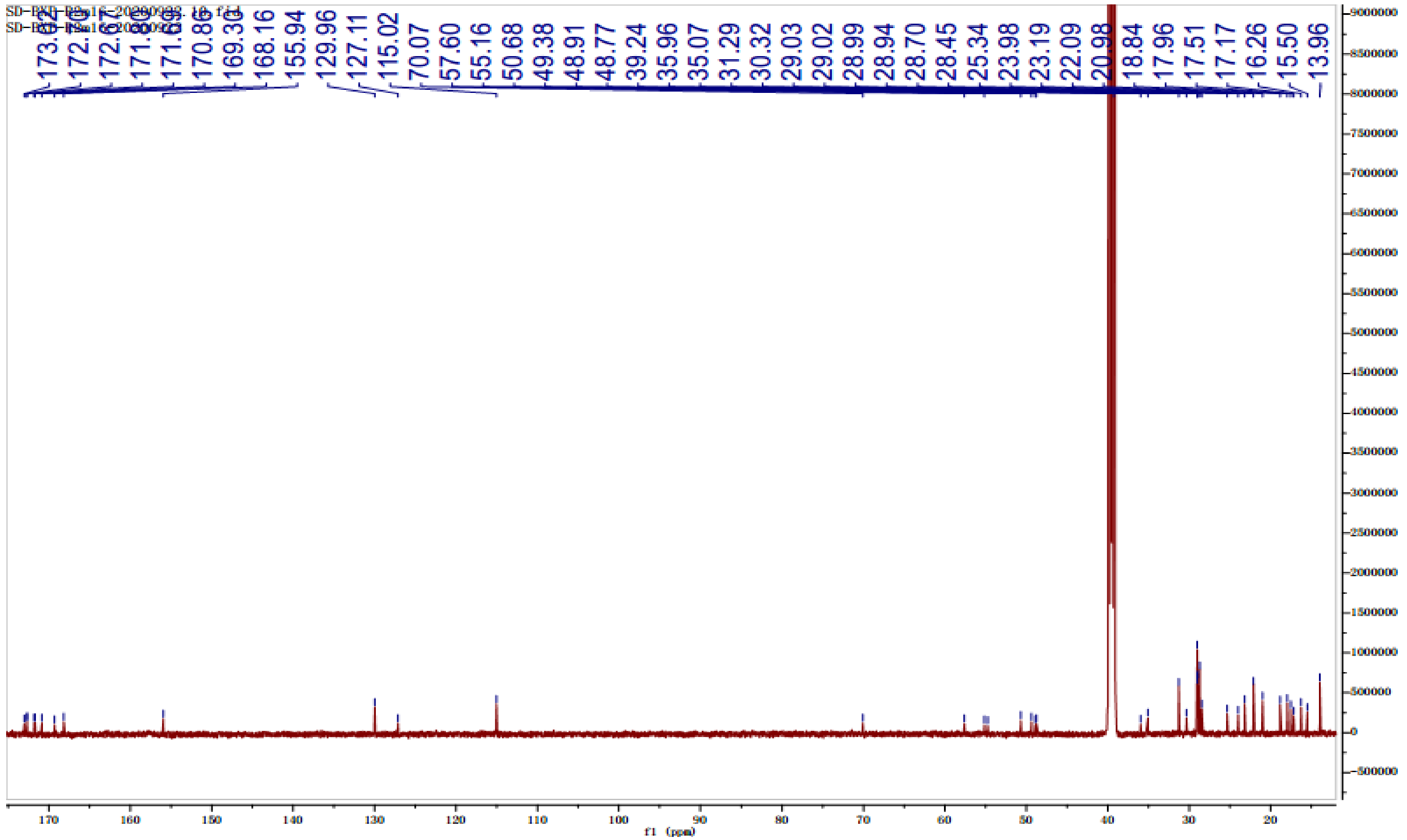

Supplementary Fig. 28. ^{13}C NMR spectrum of **1h** in DMSO-d_6

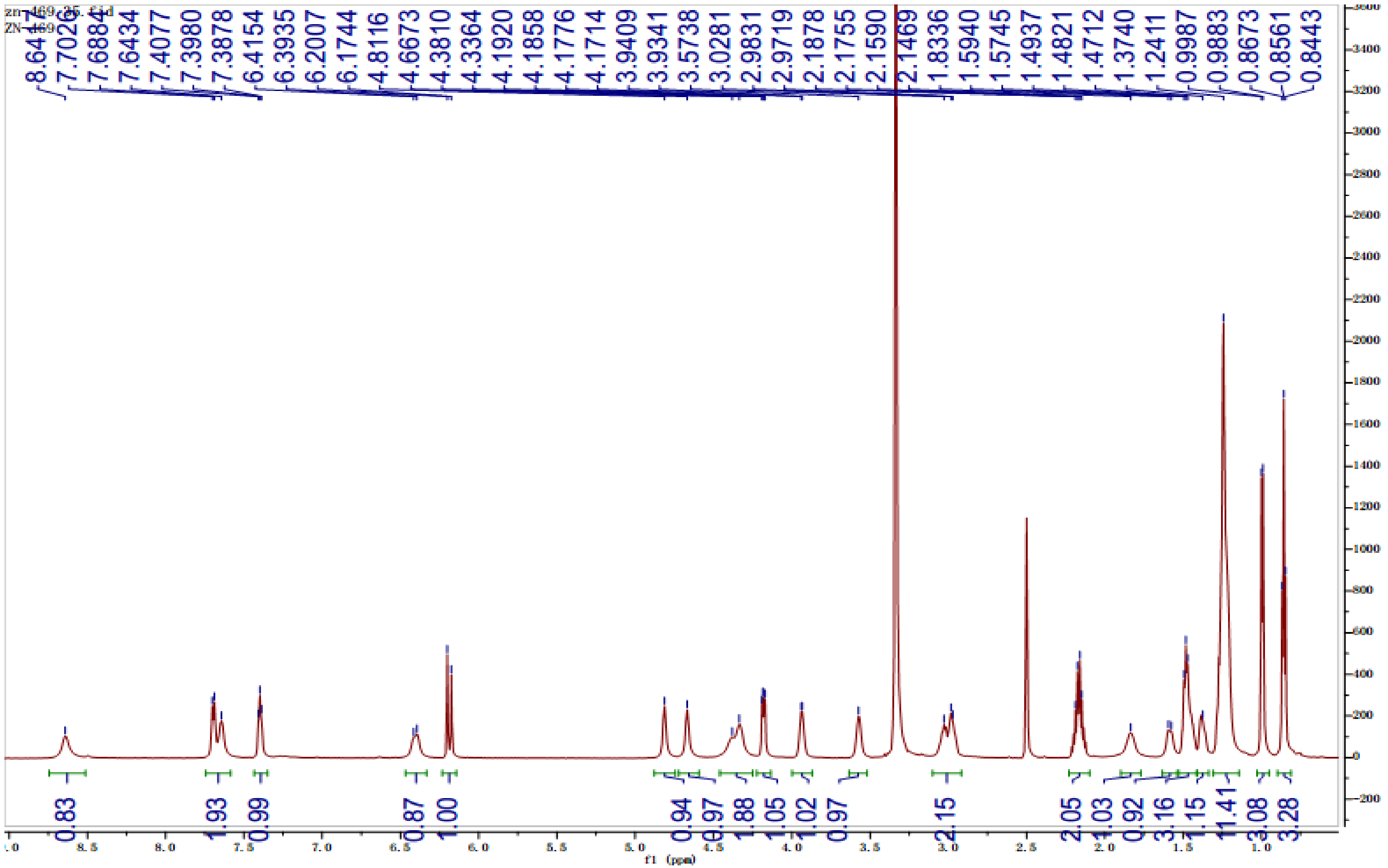

Supplementary Fig. 29. ^{1}H NMR spectrum of **7b** in DMSO-d_6

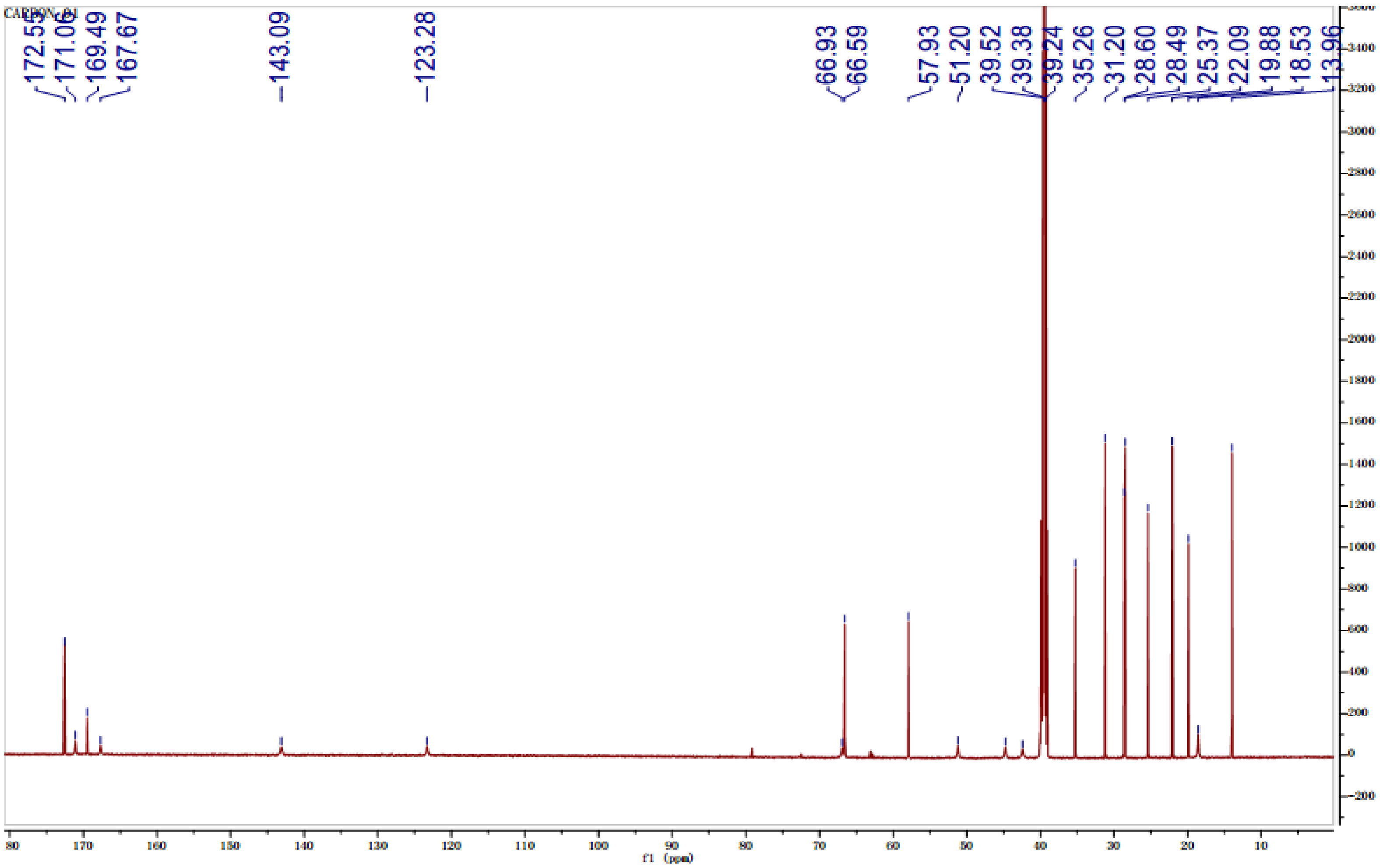

Supplementary Fig. 30. ^{13}C NMR spectrum of **7b** in DMSO-*d*6

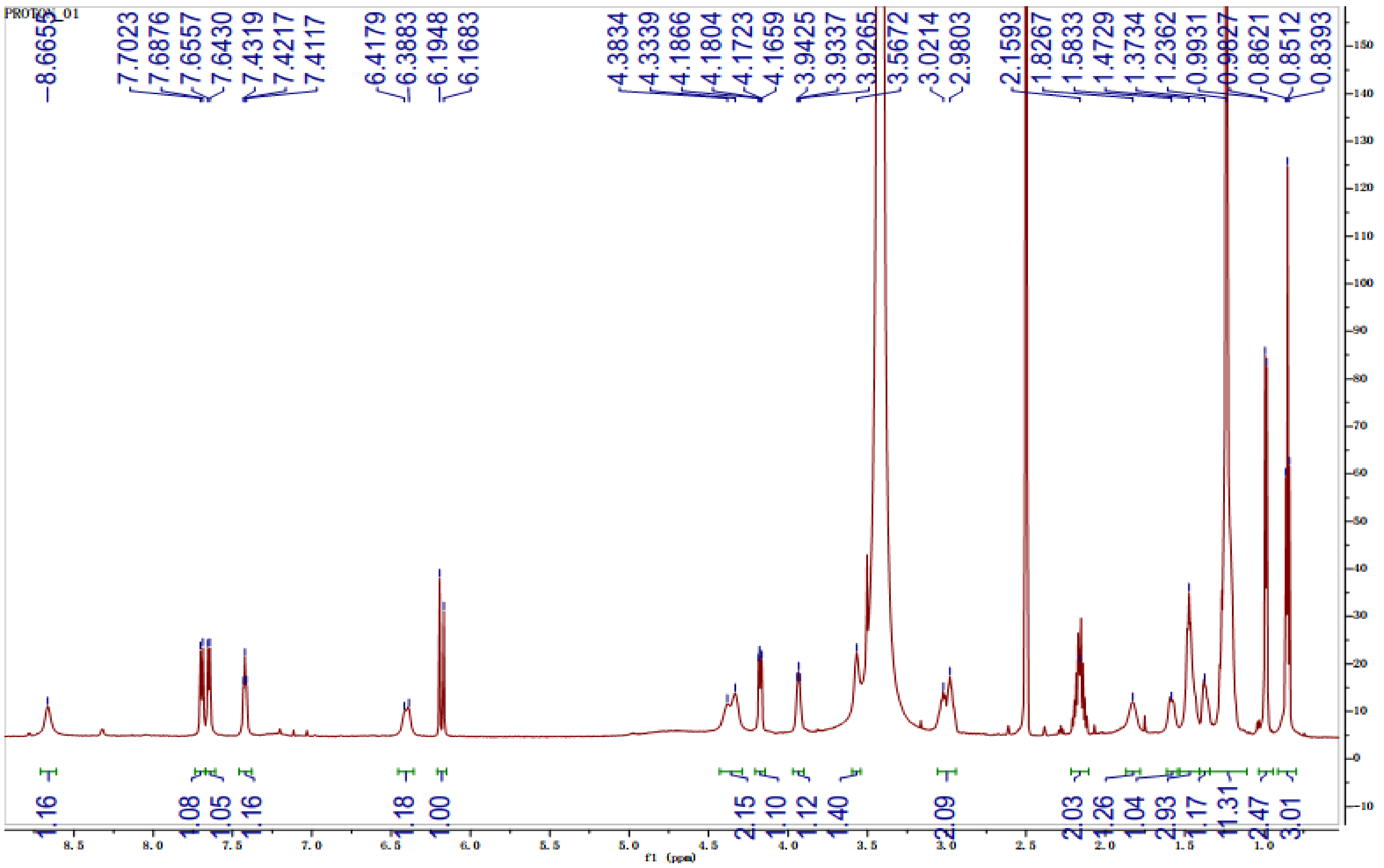

Supplementary Fig. 31. ^{1}H NMR spectrum of **7c** in DMSO-d_6

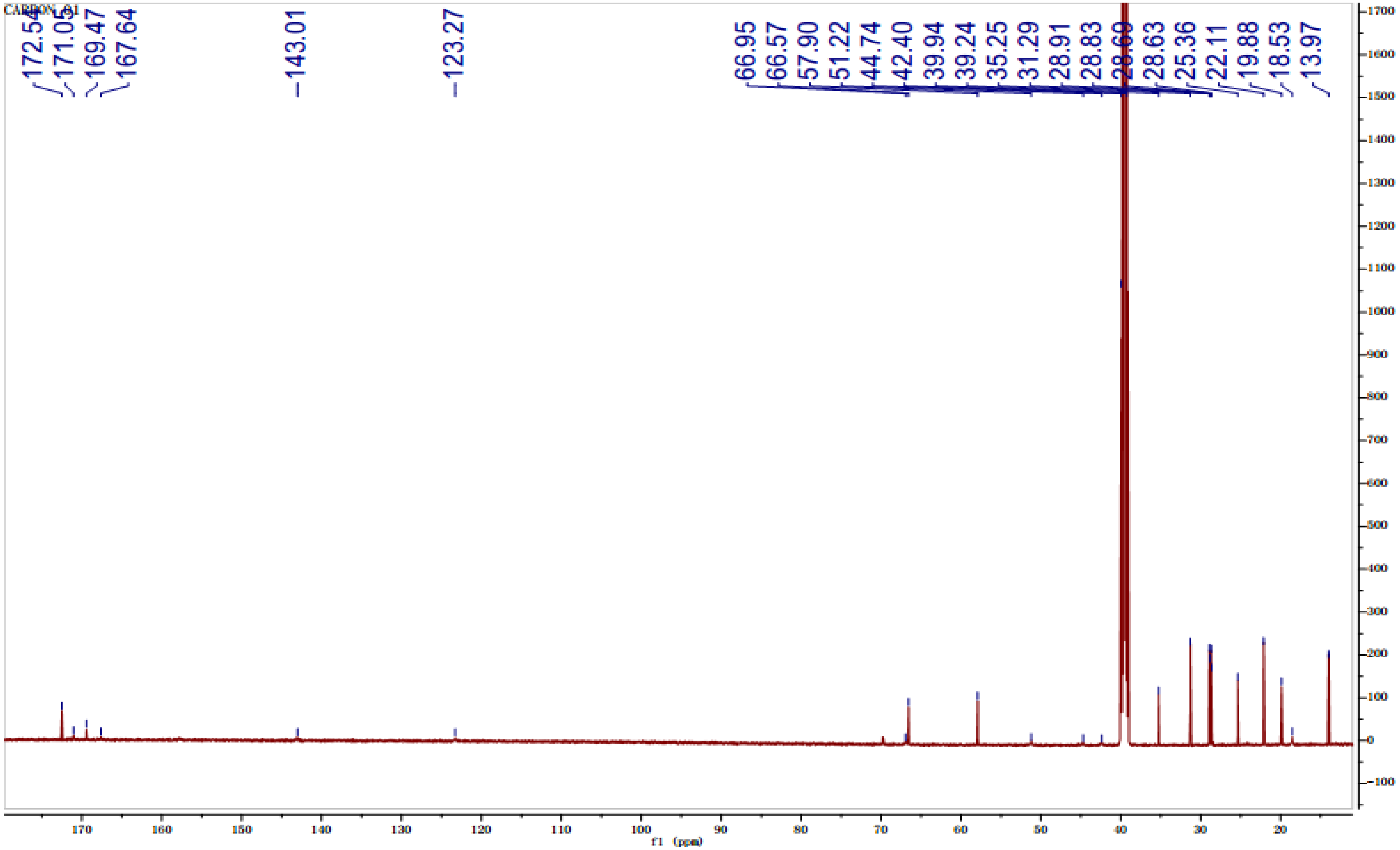

Supplementary Fig. 32. ^{13}C NMR spectrum of **7c** in DMSO-d_6
