# Supplementary material for: Engineering and elucidation of the lipoinitiation process in nonribosomal peptide biosynthesis
Source: Nat Commun. 2021 Jan 12;12:296. doi: 10.1038/s41467-020-20548-8 (PMC7804268; doi:10.1038/s41467-020-20548-8)
Supplement: Supplementary file 3 — Descriptions of Additional Supplementary Files [file 41467_2020_20548_MOESM3_ESM.docx]

Descriptions of Additional Supplementary Files

**Supplementary Data 1**

Description: Bacteria strains, Plasmids and Sequences of primers used in this study.

**Supplementary Video 1**

Description: Animation showing conformational changes of RzmA-Cs during the “unbound-bound-released” catalytic cycle. RzmA-Cs adopts a typical “V-shaped” configuration formed by its N- and C-lobes. Binding of substrates (C8-CoA and L-Leu-SNAC) requires and/or induces the two lobes to close up, with residues in the “latch” and “floor” regions (colored in brown and red) moving towards the active site. After condensation reaction, the two lobes open up a little bit, but the “floor” loop flips dramatically up against the binding site of CoA, probably accommodating the product release. Finally, for a new cycle the two lobes open more, with the “floor” loop moving back down as in the “unbound” status.
